# Towards the restoration of the Mesoamerican Biological Corridor for large mammals in Panama: comparing multi-species occupancy to movement models

**DOI:** 10.1186/s40462-019-0186-0

**Published:** 2020-01-09

**Authors:** Ninon F. V. Meyer, Ricardo Moreno, Rafael Reyna-Hurtado, Johannes Signer, Niko Balkenhol

**Affiliations:** 1 Departamento de Conservación de la Biodiversidad, El Colegio de la Frontera Sur, Lerma, Campeche Mexico; 2 Wildlife Sciences, Faculty of Forest Sciences, University of Göttingen, Göttingen, Germany; 3 Fundación Yaguará Panamá, Ciudad del Saber, Panama; 4 Smithsonian Tropical Research Institute, Balboa, Ancón Panama

**Keywords:** Landscape connectivity, Habitat suitability, Least-cost path, Movement behavior, Step selection functions, White-lipped peccary

## Abstract

**Background:**

Habitat fragmentation is a primary driver of wildlife loss, and the establishment of biological corridors is a conservation strategy to mitigate this problem. Identifying areas with high potential functional connectivity typically relies on the assessment of landscape resistance to movement. Many modeling approaches exist to estimate resistance surfaces but to date only a handful of studies compared the outputs resulting from different methods. Moreover, as many species are threatened by fragmentation, effective biodiversity conservation requires that corridors simultaneously meet the needs of multiple species. While many corridor planning initiatives focus on single species, we here used a combination of data types and analytical approaches to identify and compare corridors for several large mammal species within the Panama portion of the Mesoamerican Biological Corridor.

**Methods:**

We divided a large mammal assemblage into two groups depending on the species sensitivity to habitat disturbance. We subsequently used cost-distance methods to produce multi-species corridors which were modeled on the basis of (i) occupancy of nine species derived from camera trapping data collected across Panama, and (ii) step selection functions based on GPS telemetry data from white-lipped peccary *Tayassu pecari*, puma *Puma concolor*, and ocelot *Leopardus pardalis*. In addition to different data sources and species groups, we also used different transformation curves to convert occupancy and step-selection results into landscape resistance values.

**Results:**

Corridors modeled differed between sensitive and tolerant species, between the data sets, and between the transformation curves. There were more corridors identified for tolerant species than for sensitive species. For tolerant species, several corridors developed with occupancy data overlapped with corridors produced with step selection functions, but this was not the case for sensitive species.

**Conclusion:**

Our study represents the first comparison of multispecies corridors parametrized with step selection functions versus occupancy models. Given the wide variability in output corridors, our findings underscore the need to consider the ecological requirements of several species. Our results also suggest that occupancy models can be used for estimating connectivity of generalist species. Finally, this effort allowed to identify important corridors within the MBC (i) at a country scale and (ii) for several species simultaneously to accurately inform the local authorities in conservation planning. The approach we present is reproducible in other sites and/or for other species.

## Background

To face the deleterious impacts of habitat loss and fragmentation on biodiversity worldwide, conservation efforts have increasingly focused on maintaining and/or restoring functional connectivity among habitat fragments at landscape scales, in particular through the establishment of biological corridors [1, 2]. Biological corridors can have different purposes such as connecting habitat patches within an individual home range, or connecting large habitat areas for seasonal migration. Here we focus on corridors which are specifically designed to facilitate movement and successful dispersal of individuals between populations to increase gene flow and long-term population viability [3, 4].

Many modeling approaches exist to identify areas with high potential functional connectivity, i.e., the degree to which landscapes facilitate or impede the movement of organisms [5]. It is increasingly recognized that an understanding of animal behavior rather than expert opinion alone is of paramount importance to effectively account for environmental effects on functional connectivity [3, 6]. However, to date relatively few studies compared the results obtained with different data sources and methods for assessing connectivity (but see [7, 8]), especially in tropical forests. A common approach to model biological corridors requires to first estimate a resistance surface, i.e., a spatial layer that reflects the degree to which a location in the landscape facilitates or impedes movement of a focal species (e.g., high resistance might be assigned to a road [6, 9]). Ideally, resistance should be estimated from actual dispersal data [10], but collecting a sufficiently large sample size of such data is extremely challenging [8, 11]. Genetic data can also be used to infer successful dispersal and reproduction among populations [12], but genetic data do not directly convey how animals move across the landscape, in addition to not always being available for species of conservation concern. Hence, resistance surfaces are often derived from habitat suitability (HS) values [7, 11, 13], which can be estimated empirically using, for example, occurrence information.

Occurrence data can be obtained in many different ways [9], and several recent studies used presence point data from satellite telemetry collars [7, 8, 11, 13, 14]. An important concern with this approach is defining the availability domain (i.e., what is available to the animal [9]). Moreover, presence points collected via telemetry studies likely represent locations from relatively few individuals, hence the sample size is often low. In contrast, camera trap data analyzed in an occupancy modeling framework explicitly estimate non-detection from true absence [15], and the challenge of having to define habitat availability is lessened. This is one reason why models based on camera-trapping data may be superior in estimating resistance than models derived from presence-only data. Moreover, when the survey is robustly designed, the entire population in the area sampled is assumed to be surveyed, including non-collared animals. Although camera trap data is increasingly widespread and available, because it is often easier and cheaper to obtain at a large scale than satellite collar data, their use in estimating functional connectivity has been very scarce (but see [16]).

Several studies showed the ability of occurrence data to predict dispersal habitat and hence to provide meaningful estimates of functional connectivity [7, 13, 14]. However, a major concern is that with occurrence data, the environmental characteristics of the point locations are assessed, rather than the environment connecting the points [9]. This reflects the assumption that animal choose travel routes on the basis of the same factors they use to choose habitat, although presence at a point versus movement between points are different processes that may be driven by different factors [6]. Therefore, connectivity models based on occurrence data may not always adequately reflect movement across the landscape, and have a tendency to underestimate functional connectivity [17]. As a result, it has often been argued that connectivity models and underlying landscape resistance surfaces based on observed movement data would better capture areas facilitating the dispersal of species [6, 8–11]. Yet, despite considerable advances in technological tools, acquiring sufficient and accurate GPS locations to infer movement under dense tropical forest canopy remains both costly and challenging [18]. Gaining a better understanding of how data types perform in tropical forests is crucial for ensuring that limited resources are efficiently invested in connectivity conservation [8, 19]. For example, if models derived from occupancy/camera traps data capture the movement process as well as models derived from GPS collar data, then time-consuming and costly data collection efforts may not be necessary. However, if occupancy data perform poorly, then the effort for collaring is well justified [8].

The choice of the focal species is another subject of debate in connectivity modeling, and often depends on the availability of data [20]. Many large-scale corridor initiatives focus on a single species (e.g., Yellowstone to Yukon Conservation Initiative for grizzly bear *Ursus arctos,* Jaguar Corridor Initiative in Latin America, *Panthera onca*), also referred to as a surrogate species, because it is assumed that the needs of an entire community are addressed by focusing on the requirements of a surrogate [4]. However, the conservation of a single umbrella species, typically a large-bodied carnivore species with extensive area requirements and high mobility, might not necessarily facilitate conservation of more sensitive, less mobile, or smaller species, given that they may have very different ecological and connectivity requirements [21, 22]. As many species are threatened by fragmentation, conservation corridors may more effectively protect regional biodiversity if they are developed to support the movement of multiple species simultaneously and with the same ecological requirements, rather than movement of a single species [23, 24].

In this study, we address the issues of choosing focal species and data type by comparing a set of connectivity scenarios derived from resistance surfaces that were estimated using varying: (1) species, (2) data sources, and (3) procedures to estimate landscape resistance. Our study was focused in the Mesoamerican Biological Corridor (MBC) which is a large-scale conservation corridor extending from Southeastern Mexico to Panama. In spite of substantial financial effort invested since it initiated in the 1990’s [25], its effectiveness has been questioned for large terrestrial mammals [26, 27] including in Panama [28, 29]. This is problematic because the Isthmus of Panama is the last and narrowest portion of the MBC which connects Mesoamerica to South America, and has acted as an intercontinental land bridge for a large suite of taxa -including mammals- for millions of years [30]. Promoting functional connectivity by identifying important areas that would facilitate movement and gene flow in mammals across Panama will support ecosystem function and benefit biodiversity in general, because mammals have important functions within ecosystems [31].

We used a) detection-non detection data from camera trapping surveys, and b) empirical movement data from satellite telemetry to develop multi-species connectivity maps for two groups of medium to large-sized terrestrial mammal species that vary in their sensitive to habitat disturbance. Because species may respond differently to landscapes features, we expected the resistance surfaces and resulting connectivity scenarios to not overlap between the two groups of species. However, as previous studies showed that different data types produce resistance surfaces with similar variables and relationships to resistance [8, 32, 33], we predicted that both data types would produce qualitatively similar resistance surfaces within the same group of species.

## Methods

### Study area

**Fig. 1:**
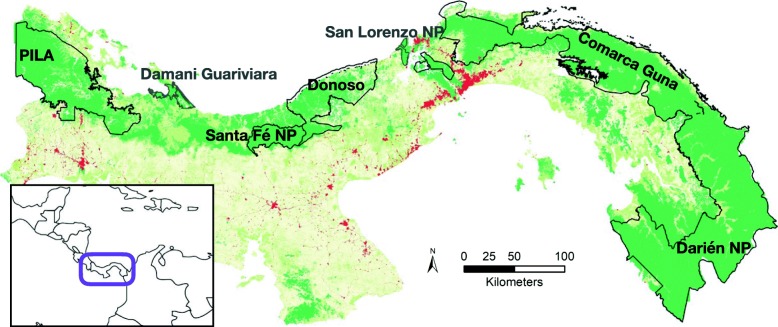
Land cover in Panama with primary and secondary mature forest (green), disturbed forest (light green), non-forest cover (beige), urban areas (red), and protected areas within the MBC (black lines). Inset: location of Panama in Central America

The s-shaped Isthmus of Panama is approximately 750 km long and 60 km wide at its narrowest part along the Panama Canal in Central Panama (Fig. 1). The MBC portion in Panama is known as the ‘Corredor Biológico Mesoamericano del Atlántico Panameño’ (CBMAP) because it overlaps with the Atlantic side of the isthmus where most of the forest remains. Panama lies in the moist tropics with a dominant vegetation that is semi-deciduous or evergreen lowland forest, or sub-montane wet forest [34]. Panama has lost 40% of its forest cover since the 1950’s mainly for cultivation and cattle pastures [35]. Today, of the 43% of land that remains forested, 44% are under protection corresponding to 22% of the country’s land area [35]. Outside protected areas (PA) the country is a mosaic of both old-growth and secondary forest patches surrounded by agriculture, pastures, and human settlements [34].

### Focal species

We used data from the nine largest terrestrial mammal species (i.e., > 12 kg) that we divided into two groups according to their sensitivity to habitat disturbance which was evaluated on the basis of expert opinion. All are mostly forest specialists species and are either herbivorous-frugivorous, i.e., Baird’s tapir *Tapirus bairdii*, white-lipped peccary *Tayassu pecari,* collared peccary *Pecari tajacu*, white-tailed deer *Odocoileus virginianus*, Central American red brocket deer *Mazama temama*; or carnivorous, i.e., jaguar *Panthera onca*, puma *Puma concolor*, ocelot *Leopardus pardalis;* or insectivorous, i.e., giant anteater *Myrmecophaga tridactyla.* White-lipped peccary, tapir and giant anteater do no longer occur in as many areas in Panama as the other focal species [36–38], are highly threatened by habitat loss and hunting for bush meat, and are typically the first to disappear with habitat disturbance. Hence, we included them in the ‘sensitive’ group. We categorized the other six species in the ‘tolerant’ group. While they are also poached for bush meat or killed in retaliation of domestic animal depredation [39] they are less sensitive to habitat disturbance, and some of them are quite vagile in fragmented landscapes (i.e., puma, [33], and white-tailed deer [40]).

### Animal locations and movement data

We used two data types in our analysis: a) Detection-non detection data were obtained from large-scale camera trapping surveys scattered across Panama (see [41] for details, Additional file 1); b) GPS telemetry data were obtained from white-lipped peccaries (two females and a male), and a puma (male) that we captured between 2016 and 2018 in the Darién forest in eastern Panama. They were fitted with an iridium GPS collar unit (TGW-4570-4) equipped with a CR-2A automatic release mechanism (Telonics, AZ, USA). The white-lipped peccary is a social species that lives in large herds. As the individuals were from different herds, they each represented the movement of an entire group [41]). We also captured and fitted an iridium GPS collar (Vectronic Aerospace GmbH, Germany) to a male ocelot in August 2017 in Soberania National Park (NP) in Central Panama. All procedures followed standard protocols approved by the Ministry of Environment of Panama (permit No. SE/A-104-15), and the Research Ethics Committee of El Colegio de la Frontera Sur, Mexico (CEI-O − 068/16). The GPS collars were programmed to get a fix every hour during 14 months. Due to the lack of signal from the collars after the release date and the rugged terrain, we could not recover the collars to extract all the data stored on-board. All individuals showed home ranging behavior when using the semi-variance approach developed by Fleming et al. [42], (see [41] for the white-lipped peccary). We used data from white-lipped peccary as a proxy for the sensitive group, and data from puma and ocelot as a proxy for the tolerant group.

### Environmental variables

We tested the influence of six environmental covariates on the probability of occupancy and movement of the focal species (Table 1). Variables were chosen on the basis of literature and opinion of experts [29]. We used 30 m as the spatial grain size for all variables, and generated raster layers in ArcMap (v.10.3.1 ESRI, California). All layers were obtained from the Ministry of Environment of Panama (MiAmbiente), except for forest loss and forest cover for which we used freely available high resolution global maps [43]. Since animals may respond to different environmental features at different scales, using a single scale for all the variable may result in inaccurate estimates of landscape resistance [10, 33]. Therefore, we first determined the most appropriate scale for three variables (i.e., village, loss and forest cover) via a univariate analysis, to further combine the results in a multi-scale model of habitat suitability (Table 1; Additional file 2). We centered and scaled the covariates [44], and we performed a Spearman correlation test to avoid multicollinearity (defined here as rho > |0.6|).

**Table 1:** Environmental variables tested in the habitat suitability models

Variables	Code	Scale tested (radius around each location point)
Distance to nearest road	road	
Density of human settlements	village	2 km, 5 km, 10 km, 20 km
Forest loss	loss	150 m, 500 m, 1 km, 2 km
Distance within protected area	DWPA	
Elevation	elevation	
Forest cover	FCOV	150 m, 500 m, 1 km, 2 km at a forest threshold of 50, 75, 90%

#### Data analysis

**Fig. 2:**
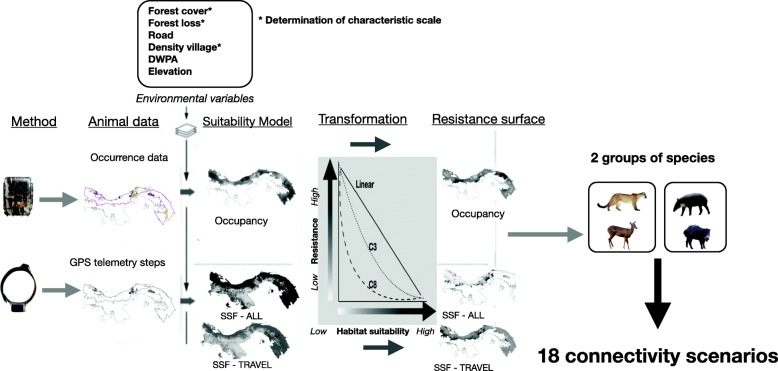
Workflow chart to estimate landscape resistance from each data type and create multi-species connectivity scenarios for two groups of species. A suite of suitability models were developped by integrating environmental variables and by using (1) occupancy modeling or (2) step selection functions (SSF). Each suitability model was then predicted across our study area which was the MBC in Panama. Three negative functions (one linear and two exponential) were used to transform the habitat suitability (from occupancy) or suitability for movement (from SSF) to landscape resistance. Each of the 18 landscape resistance surfaces was subsequently used as input for connectivity modeling. Diagram adapted from [8]

To design multi-species connectivity scenarios and identify wildlife corridors for each of the two groups of species, we first developed habitat suitability models by estimating the probability of occupancy using camera trapping data, and movement suitability models by calculating the probability of movement through step selection functions using GPS telemetry data. We then transformed the habitat suitability and suitability for movement values into resistance values. The resulting resistances surfaces were used as input for mapping functional connectivity across the MBC in Panama. The workflow we followed is presented in Fig. 2.

### Modeling habitat suitability using occupancy and movement data

We conducted a two-step conditional logistic regression to quantify selection for each habitat attribute at the appropriate scale [7, 8, 10]. In a conditional logistic regression, used habitat is compared to available habitat, conditioned on the current position. We estimated the probability of occupancy for each of the nine focal species from detection-non detection data obtained via camera trapping, and by using the multi-species hierarchical occupancy model in a Bayesian framework that was described by [45]. This model estimates species-specific parameters as random effects of a community level distribution which is particularly advantageous for rare species such as jaguar, giant anteater, tapir and white-lipped peccary (see [29] for details). The occupancy model took the form:

$$ \mathrm{logit}\ \left({\Psi}_{\mathrm{i}\mathrm{j}}\right)={\upalpha}_i+{\upalpha}_{\mathrm{i}1}\ast \mathrm{V}1+{\upalpha}_{\mathrm{i}2}\ast \mathrm{V}2+\dots +{\upalpha}_{\mathrm{i}\mathrm{n}}\ast \mathrm{V}\mathrm{n} $$

where Ψ_ij_ was the probability of occupancy of species *i* at camera site *j*, α_*i*_ was the intercept of the model specific to species *i*, and α_in_ was the coefficient of variable Vn specific to the species *i.*

We also developed step selection functions (SSF) to estimate suitability for movement from the GPS telemetry data set. A SSF compares the covariate values at the end point of observed steps (i.e., steps that the animal actually made) with covariate values at the end of control steps (steps that were deemed available to the animal but unused). A step was defined as the straight-line path between two consecutive GPS fixes, here with a sampling rate of 1 hour. Using the R Package ‘amt’ [46], landscape feature availability was estimated by generating 100 random steps (calculated using a gamma distribution, see [46]) which were compared with the observed ones. Observed and random steps shared the same starting point, but differed in their length and angular deviation.

As environmental variables may confer different levels of resistance to different types of behavior (i.e., traveling, stationary), failing to consider an animal’s behavioral state may be insufficient in determining habitat selection during dispersal, and hence may result in misidentification of wildlife corridors [8, 11]. When no dispersal data is available, habitat selection measured during directed movement states (or traveling) may provide a reliable proxy to infer functional connectivity [8, 11]. We therefore developed SSF to quantify resource selection for a combined model which included all available data, SSF-All, and for a traveling model which included only traveling data, SSF-Travel. To focus on traveling behavior, we excluded steps < 100 m, < 150 m, and < 200 m for the ocelot, white-lipped peccary, and puma respectively. Turning angle is also sometimes used to separate movement behavior, i.e. low turning angles steps are classified as travel behavior (e.g., [33, 47]). However, when following the groups of white-lipped peccaries for several days, we noticed that even when they were moving fast (hence traveling), they sometimes took very sharp angle (> 90°). Tapirs are also known to walk in a zigzagging manner [48, 49]. Relying on turning angles to determine the movement mode could therefore be misleading for some of our focal species, so we decided to not take it into account.

Since individuals might respond to the environmental covariates differently, it is common practice to use either mixed effects models with individuals as random terms [50], or to average individual coefficients for obtaining coefficients at the population level [51]. However, with high individual-level differences and relatively small sample size, this approach could lead to overgeneralization and spatial biases [52]. Therefore, we developed a SSF for each individual [52] by testing a set of candidate models that included additive uncorrelated covariates as main effects. The best supported model was selected using AICc [53]. We used the coefficients of the best supported SSF models to create surfaces of suitability for movement along the CBMAP for each individual, and for each behavior (‘All’ and ‘Travel’). As in Keeley et al. [7] the value of suitability for movement of each cell was calculated as:

$$ \mathrm{S}={\upbeta}_1\ast {\mathrm{V}}_1+{\upbeta}_2\ast {\mathrm{V}}_2+\dots +{\upbeta}_{\mathrm{n}}\ast {\mathrm{V}}_{\mathrm{n}} $$

where S was the suitability for movement and β_i_ was the coefficient for the variable V_i_.

We rescaled all the movement suitability maps from SSF to a range of 0–1 with the equation:

$$ f(x)=\frac{\left(x-\min \right)}{\max -\min } $$

where x was the value of suitability for movement of a grid cell, and min and max were the minimum and maximum values of suitability for movement of the suitability for movement surface. Values near 1 indicated the most suitable conditions for movement, while values near 0 indicated the least suitable habitat for movement.

### Estimating the resistance

It is generally accepted that resistance is the negative inverse of habitat suitability (Fig. 2 [4, 9, 54]). It is also increasingly recognized that during dispersal or prospecting movements, animals may move more readily through lower suitable habitat such that resistance increases only moderately as suitability decreases from its maximum value, and then increases dramatically at lower suitability values [7, 13, 54, 55]. Hence, we tested three transformations to translate habitat suitability into resistance: a negative linear transformation,

$$ \mathrm{R}=100-\left(100\ast \mathrm{HS}\right) $$

and two negative exponential transformations which assigned high resistance values to the lowest habitat suitability values, following the equation developed by Trainor et al. [54]:

$$ R=100-99\frac{\left(1-{e}^{\left(-c\ast HS\right)}\right)}{1-{e}^{-c}} $$

where R was the resistance, HS was the habitat suitability (i.e., the occupancy probability ψ, or the probability of movement S as derived from SSF), and the factor c (3 or 8) determined the shape of the curve (Additional file 3).

Using this transformation, we developed a) species-specific resistance maps based on the occupancy output for each of the nine focal species, and b) individual-specific resistance maps with the best supported SSF models specific to each individual and each movement mode, ‘All’ and ‘Travel’.

### From single to multi-species connectivity scenarios

In order to quantify resistance for a combination of species and identify the multi-species connectivity scenarios, we standardized the unscaled resistance surfaces generated for each species (using occupancy), and each individual (using SSF). We subsequently averaged the standardized scores into combinations of sensitive and tolerant species with the raster calculator in ArcGIS (v.10.3.1 ESRI, Redlands, California). We generated 18 resistance surfaces (two groups of species, three types of data, three transformations) that ranged from 1 (lower cost) to 1000 (higher cost).

At this stage, we assigned roads and urban areas a resistance value of 85 and 95% of the maximum resistance estimated for the tolerant and sensitive groups respectively because they present a major barrier for the movement of our focal species [56]. We used the resulting resistance surfaces as input to build functional connectivity networks among the core areas using least-cost path (LCP) and circuit theory methods. The LCP approach estimates the shortest distance between target core areas while accounting for resistance to movement [57]. Circuit-theory connectivity is based on random walk and uses the principles of an electric circuit where a current (animal) flows through nodes (habitat patches or cores) connected by resistors (landscape matrix) with voltage (probability of animal travel) and resistance (permeability of matrix). The resulting product is a prediction of ‘current density’ or a probability of movement across each pixel of the landscape [58]. We implemented the analysis in Linkage Mapper (v2.0.0 in ArcGIS 10.3.1; [59]). We used the PinchPoint Mapper tool and the All-to-one mode to estimate resistance values within least-cost corridors in order to identify and map pinch points (i.e. bottlenecks) within the resulting corridors. Given the relatively large spatial requirement of the study species, we used a cost-weighted distance cutoff of 25,000 to buffer our least-cost path so corridors had a biologically meaningful width of at least 1 km at their bottleneck.

### Defining areas important to connect

Linkage Mapper requires to specify the areas between which to estimate functional connectivity and establish corridors. Intuitively, one could contemplate using the protected areas, but because not all protected areas in Panama still have populations of all study species [36], or conversely species populations could occur in non-protected areas, this approach would lead to inaccurate results. Instead, similar to Hofman et al. [56], we used the output of the occupancy analysis to determine habitat concentration area (henceforth core area) defined as areas known to harbor important populations of the focal species [60]. We plotted the probability of occupancy against the proportion of the study area (Additional file 4). We identified the occupancy threshold where the slope was the highest, and used this occupancy value as the threshold to identify areas where occupancy was at least equivalent or higher to that value. The proportion of area which was considered suitable and which we hence sought to connect was larger for the tolerant group (50% of the study area, 8 core areas; ψ = 0.2) than for the sensitive group (area = 40%; 6 core areas; ψ = 0.3). This seems intuitively correct given that sensitive species are not as widely distributed in the study area compared to the more tolerant ones. We cross-checked the output maps of core habitats for the focal species (Additional file 5) with our opinion and previous studies of species distribution model [27, 37, 38].

## Results

### Animal locations and scale of analysis

We obtained 5315 unique detections of the nine focal species during 43294 camera trap nights. We also acquired 3400 GPS fixes from the sensitive group, and analyzed 3098 observed steps of which 1133 were classified as ‘traveling’ mode. We received 2682 GPS fixes for the tolerant group, and analyzed 2311 observed steps of which 759 were classified as ‘traveling’ mode (Additional file 6).

The AICc ranking of the occupancy and movement models showed that the scale of response varied between the two data sources, among individuals, and whether all the data or only the travel data were used (Additional file 7). The best scale for the forest cover varied the most with no clear pattern for its threshold, but the sensitive group tended to respond to forest cover within a larger area (up to 1 km) than by the tolerant group (150 m). Likewise, the scale of forest loss varied substantially (from 150 m to 2 km) with no clear selection pattern. The scale for density of village also tended to vary between individuals and between data type. However, it remained the same within each individual when using ‘traveling’ and ‘all’ data, except for the ocelot and a white-lipped peccary. In general, sensitive species responded to anthropogenic variables (i.e., road and village) at a smaller scale than tolerant species, whereas they responded to forest cover at a larger scale than tolerant species.

### Occupancy and movement models

Our occupancy model included all variables but forest loss. The sign and intensity of the variables affecting occupancy differed by species (see [29], Additional file 8). Occupancy of all species but puma tended to increase deeper inside the protected areas, especially white-lipped peccary, white-tailed deer and collared peccary. Most species, but in particular the white-lipped peccary, responded positively to forest cover. The relatively small and non-significant coefficients of density of villages and distance to roads reflect their little effects on the occupancy of most species.

The covariates included in the highest-ranking step selection models remained relatively consistent across movement behavior and individuals (Table 2, Additional files 9 and 10). Forest loss was retained in all the best step selection models, and forest cover too with the exception of puma. The sign of the relationship, indicating preference or avoidance, changed for some variables between individuals and data source (Table 3). Although the sign changed little with movement behaviour (‘All’ versus ‘Travel’), its strength varied but not in a consistent manner. In general, when traveling, the strength of selection for forest cover was higher, and the strength of selection for forest loss lower than when pooling all the relocation data (Table 2). The sensitive group had a tendency to roam at higher elevation, while the tolerant group selected areas at lower elevation. All the species remained deeper inside the protected areas expect for puma, and had a tendency to select forest loss. Road had little influence on the animals, as evidenced by the very small coefficient.

**Table 2:** Best supported step selection models developed for each individual and using two behavioral movement modes (see Additional file 10 for standard error and confidence intervals)

	Mode	Model
TOLERANT		
*Puma*	SSF-All	-0,26*elevation + 0,47*loss + 0,1*FCOV + 1,22*DWPA
	SSF-Travel	- 0,56*elevation + 0,28*loss + 0,09*FCOV + 0,68*DWPA
*Ocelot*	SSF-All	0,62*FCOV - 0,14*village + 0,03*loss – 0,32*DWPA – 0,02*elevation − 0,01*road
	SSF-Travel	0,82*FCOV - 0,14*village - 0,16*loss – 0,71*DWPA + 0,10*elevation
SENSITIVE		
*WLP1*	SSF-All	−0.13*road + 0,63*elevation + 0,03*loss - 0,06*FCOV + 0,09*village
	SSF-Travel	− 0,08*road + 0,62*elevation + 0,01*loss – 0,09*FCOV + 0,17*village
*WLP2*	SSF-All	1,15*DWPA + 0,44*FCOV + 0,17*loss
	SSF-Travel	−1,61*village + 0,65*FCOV + 0,15*loss
*WLP3*	SSF-All	0,60*elevation - 0,46*DWPA + 0,20*FCOV + 0,08*loss + 0,20*village
	SSF-Travel	0,60*elevation − 0,09*DWPA + 0,28*FCOV - 0,18*loss

Estimates < 0 indicate avoidance or unsuitability of habitat for movement, whereas estimates > 0 indicate selection or suitability of habitat for movement. WLP stands for white-lipped peccary

**Table 3:** Relationship between habitat suitability and six environmental variables for each individual or species. Suitability models were developed with different data types, (a) detection-non detection data analyzed in an occupancy modeling framework (ψ), and movement data analyzed with step selection functions and based on different movement behavior, (b) all data (SSF-All) and (c) traveling data only (SSF-Travel)

Species		ROAD	VIL	LOSS	FCOV	DWPA	ELEV
*Puma*	ψ	_	+		–	+	–
	SSF-All	0	0	+	+	+	–
	SSF-Travel	0	0	+	+	+	–
*Ocelot*	ψ	+	+		+	–	+
	SSF-All	–	–	+	+	–	–
	SSF-Travel	0	–	–	+	–	+
*WLP*	ψ	+	+		+	–	–
	SSF-All	-^a^	+	+	+^b^	+/−	+
	SSF-Travel	-^a^	+/−	+^b^	–	–	+

A zero indicates that the variable was not retained in the best supported model. ^a^One individual showed this pattern while the variable was not retained in the best supported models of the two other animals, ^b^Two individuals out of the three monitored showed this pattern. WLP stands for white-lipped peccary. See table 1 for variable code

### Multi-species connectivity scenarios

**Fig. 3:**
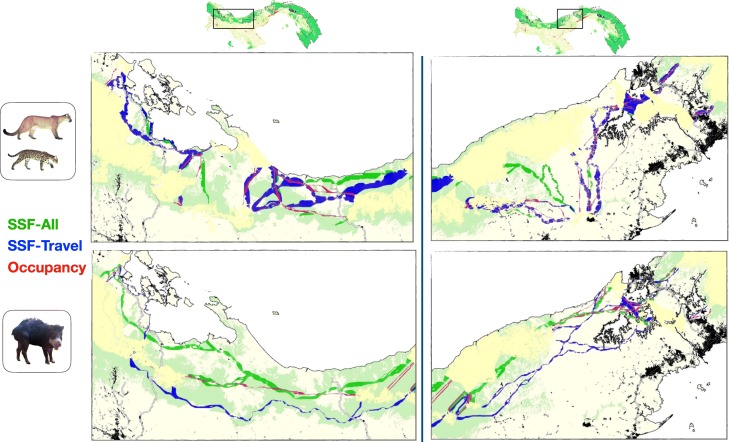
Multi-species connectivity scenarios developed to connect core areas for large mammals in Panama. Corridors were developed in the Western part of Panama between the Amistad International Park and the Santa Fé NP-Donoso block (left maps), and in Central Panama (right maps), for two distinct groups of species that were considered tolerant (represented by ocelot and puma) or sensitive to habitat disturbance (represented by white-lipped peccary). These connectivity models were derived from resistance surfaces estimated through step selection functions using all the relocation data (green), step selection functions using relocation data during travel movement (blue), and occupancy modeled at the community level for nine mammal species (red), and using the negative exponential transformation curve (c8). Urban areas are black, and main roads are the black and white lines. See Additional file 11 for maps comparing corridors modeled with varying transformation curves, data type and species

As habitat suitability models varied among data source and group of species, the resulting multi-species connectivity scenarios were also different (Fig. 3; Additional file 11). Corridor paths were always different between the two groups of species whether SSF or occupancy were used. Corridors of sensitive species usually passed through mountainous areas. When using traveling data, the corridors identified for the tolerant species were wider than corridors of sensitive species, which reflects a lower landscape resistance to movement of tolerant species than sensitive species. There was no notable difference of corridors widths when using the other data.

In Western Panama, output corridors of tolerant species were more numerous when using SSF-Travel than when using SSF-All and occupancy. Many of the corridors for tolerant species that were developed on the basis of occupancy overlapped with corridors identified with SSF. However, some corridors based on SSF were identified to pass along the Atlantic coast whereas this was not the case when using occupancy. When using SSF-All, corridors were passing through more forested areas while it was not necessarily the case when using the two other types of data. There was no such difference in Central Panama as most corridors overlapped. Corridors developed with SSF-Travel were much larger than the other corridors.

The corridor paths delineated for sensitive species with different data sets differed widely. Corridor based on SSF-Travel passed through forested areas while corridors modeled on the basis of occupancy and SSF-All were more directional. There was a corridor identified for sensitive species when using occupancy data and SSF-Travel data in the northern part of Central Panama and passing through a heavy urban area, which was not identified when using all the GPS data (i.e. SSF-All). In contrast to the tolerant species, corridors developed with SSF-All were wider than the other corridors.

The type of negative transformation (linear and exponential) had little effect on the output corridor paths for tolerant species (regardless of the data type), and on corridor paths that were modeled with occupancy data for sensitive species (Additional file 11). The only notable difference was the larger width of corridors delineated with a c8 transformation because resistance values to movement of species was lower. In contrast, output corridor paths modeled with SSF of sensitive species varied with the different transformation curves, especially when in traveling mode.

## Discussion

We compared multi-species connectivity scenarios across Panama for two groups of mammal species by using large-scale camera trapping data and GPS telemetry movement data, and a set of analytical procedures and transformation curves to estimate resistance surfaces.

### Multi-species scenarios

As expected, our results showed that connectivity scenarios differed depending on the focal species used to parameterize the resistance surface, and this regardless of the analytical approach. In the western part of Panama, the tolerant group was predicted to move with higher intensity along the Atlantic coast. In contrast, the path that would better facilitate movement of the sensitive species was predicted to pass through the Cordillera Central of Panama, most likely because areas at higher elevation are more remote and less disturbed by human activities compared to lowland areas near the coast. These findings corroborate our assumption that the Baird’s tapir, giant anteater and white-lipped peccary, which are among the most sensitive species, show a different habitat selection pattern, often less riskier than other more generalist species such as wildcats. Specifically, sensitive species were more strongly associated with larger forest cover habitat in mountainous areas, most likely to avoid riskier areas with higher deforestation and human encroachment. Although the core areas we sought to connect differed slightly between the two groups of species, the corridors identified for tolerant species were more numerous, whichever type of data we used to model them. These results indicate a larger suite of possible paths when moving between core areas, and a greater flexibility and adaptability in the matrix. Moreover, the larger width of corridors parametrized with movement data of tolerant species compared with tolerant species’, reflects a lower resistance of the matrix to movement of tolerant species.

The different connectivity scenarios are the results of habitat suitability models or models of suitability for movement, and thus reflect a habitat selection and impact of anthropogenic factors which varied among species. The species sometimes displayed contrasting patterns in the selection of habitat characteristics. For instance, the puma selected lowland areas with less forest cover, while it was the opposite for the sensitive species whose selection for forest cover was stronger and had a tendency to remain further inside protected areas. Hence, our results highlight the importance of considering multiple species with different ecological requirements to effectively estimate functional connectivity, and raise the issue of numerous past connectivity studies which focused on a single, generalist species. For example, the MBC was originally called ‘Paseo Pantera’, (Path of the Panther in English), because it was designed for jaguar [61]. Nowadays, jaguar is still often the main focal species in habitat protection and connectivity studies (e.g., [62]), given their large area requirements, high mobility, and funding potential as charismatic species. Nevertheless, our study highlights that the effectiveness of carnivores as connectivity umbrellas in tropical forests may fail to conserve community connectivity for threatened species such as the Baird’s tapir and white-lipped peccary, similarly to what previous studies found in other ecosystems [21, 22, 63]. Our results support the conclusion that highly sensitive species should be prioritized as the most important focal species for design of multi-species corridors, because less sensitive species which are often habitat generalists can more easily move through landscapes conserved for habitat specialists, whereas the opposite may not be true [4, 7, 22].

### Effect of data source

Second, our prediction that both data types would produce qualitatively similar resistance surfaces with many of the models having the same variables influencing the resistance was for the most part supported. Most models included forest cover, forest loss and elevation. Despite qualitative similar models, the choice of data type had an influence on the resulting predictions of connectivity because the sign of the relationship, and/or the strength of selection or avoidance to these variables was different. This outcome was especially striking with the corridors modeled for sensitive species, as none of the analytical approach resulted in the same corridors in western Panama. In contrast, several corridors for tolerant species that were modeled with occupancy data and step selection functions overlapped. These findings suggest that non-invasive sampling with camera traps can provide useful data for estimating functional connectivity at landscape scale, and be as informative as movement data from GPS collars to detect corridor paths for generalist species. This may especially be true when camera-trapping sampling design are spatially widespread and cover habitats with a gradient of disturbance like was our case. We did not test our corridors against dispersal data, but studies showed that models based on point data, e.g., resource selection function, are able to predict species habitat use during dispersal for leopard *Panthera pardus*, a wide-ranging carnivore [14] and for kinkajou *Potos flavus*, an arboreal mammal species [13]. Nevertheless, other studies found that resistance estimates from empirical movement data (e.g., SSF) were more similar to resistance estimates from dispersal movements, compared to resistance estimates from point data [8, 11].

A notable outcome from our analysis using the GPS telemetry data is the differences in habitat preference displayed by most individuals when traveling compared to when behavioral state was not considered. This was especially the case for sensitive species for which, and in contrast to our expectations, the SSF models revealed a smaller tolerance of animals to human-modified landscapes when traveling. In particular, when traveling, the strength of selection for forest cover was higher, while it was lower for forest loss. An opposite pattern, i.e., greater tolerance to human disturbance when traveling, was reported for carnivore species in other ecosystems (e.g., African wild dog *Lycaon pictus* [64]; lion *Panthera leo* [10]).

### Limitations and suggestions

A limitation of our study relies in the relatively restricted number of species and individuals used to parametrize the movement models in spite of considerable effort to collect data over a 2-year period. A further limitation is the lack of observed dispersal paths to validate our models. These limitations highlight the challenges associated with capturing animals and collecting long-distance movement data to evaluate functional landscape connectivity. Testing our connectivity scenarios against genetic data would provide valuable insights on landscape permeability and accuracy of the corridors, because gene flow reflects both successful movement and reproduction [8, 12, 17]. Landscape genetics is also particularly useful for large-scale assessment [65] such as was our study, but genetic data are not yet available in our study area. This said, a shortcoming when using this approach is that connectivity estimates derived from genetic usually reflect past landscape permeability and may not capture current movement and gene flow in a rapidly evolving environment such as Panama [11].

### Implications for long-term conservation of mammals in Panama

Panama is a biodiversity hotspot and has long served as a vital habitat corridor between Mesoamerica and South America for broad-ranging neotropical forest species [30]. However, this important linkage between continents is increasingly put in jeopardy by deforestation, human disruption and urban development which impede movement and most likely gene flow of several species [28, 66]. Thus, it is critical to identify areas that can facilitate the movement of multiple species within the Isthmus. While our findings show that an accurate understanding of how animals move through their environment is important for the success of corridor design, it is sociopolitical and economic considerations that will allow the protection of these corridors. For example, one of the corridor that was identified with occupancy data and SSF-Travel for the sensitive group is not realistic given that it borders a large city (Colón), where poaching pressure is very high (pers. obs.). The likelihood that tapirs and white-lipped peccaries use this path and survive is very low. Another corridor that was identified for tolerant species and which effectiveness may be uncertain, is along the Atlantic coast in western Panama. Current construction of a road stretching from the northern end of the Panama Canal all the way to the west near Costa Rica, and which is associated with real estate development, willmost likely hamper the success of the corridor.

Moreover, our modeling exercises sought to connect suitable patches, thereby implying that all the core areas were equally good in harboring healthy populations of the focal species. However, several development projects such as mines, dams, and more roads threaten the biodiversity in these core areas, especially in Santa Fé NP and Donoso. We therefore stress the importance of assessing the impacts of such projects on wildlife connectivity and take adequate measures to mitigate them. It is important to keep in mind that the lack or very small population of some sensitive species in several protected forests, i.e., Damani Guariviara or San Lorenzo NP, does not make these areas unimportant for the long-term conservation of species. They serve as stepping stones between core areas that harbor functional populations as evidenced by least-cost corridors that traverse them.

Finally, poaching remains a significant threat for wildlife in our study region (pers. obs.), especially for dispersing individuals which are key in maintaining gene flow between core populations [12]. Successfully translating this connectivity research into habitat conservation and/or restoration actions will require partnering with the competent authority for land management and planning, but also engaging other partners such as private landowners, corporates, and local indigenous communities to promote active protection of the forests and its biodiversity in general [67].

## Conclusion

Our study provides a framework to model wildlife corridors by combining different types of empirical data for multiple species simultaneously. It represents the first effort to estimate functional connectivity and identify optimal corridor locations to facilitate the movement of a suite of mammal species across an entire country in Latin America. Our findings highlight that the focal species, the data source, the analytical approach, and sometimes the transformation curve all influence the resulting connectivity scenarios. Therefore, and given the wide variety of methods employed in connectivity studies, efforts to test corridors designed are crucial (e.g., [68, 69]). Although we were not yet able to test the performance of the corridors modeled, all our multi-species connectivity scenarios show that it is critical to focus on the protection of forest at the landscape level in order to support the long-term movement of large mammals across the Isthmus of Panama. Finally, camera trapping data analyzed in an occupancy framework seems promising for estimating functional connectivity for generalist species, providing a cheaper and logistically less challenging method to telemetry.

## Supplementary information

**Additional file 1.** Methods - Locations of camera traps used to conduct occupancy modelling across Panama.

**Additional file 2.** Methods - Environmental variables and selection of characteristic scale.

**Additional file 3.** Methods - Transformation curves used to translate habitat suitability values into landscape resistance values.

**Additional file 4.** Methods- Probability of occupancy and proportion of the study area.

**Additional file 5.** Methods - Core areas of two groups of species.

**Additional file 6.** Results - GPS relocations and steps for each individual.

**Additional file 7.** Results - Determination of the optimal scale for forest cover, forest loss, and density of villages.

**Additional file 8.** Results - Species-specific coefficients for environmental variables estimated with occupancy modeling.

**Additional file 9.** Results - AICc of SSF models for each individual.

**Additional file 10.** Results - Estimates of the best supported SSF model for each individual.

**Additional file 11.** Results - Maps with multi-species connectivity scenarios.

## Data Availability

Movement data were uploaded on Movebank (www.movebank.org).

## Abbreviations

CBMAP: Corredor Biológico del Atlántico Panameño
MBC: Mesoamerican Biological Corridor
NP: National Park
SSF: Step selection functions
