# Correction to: Towards the restoration of the Mesoamerican Biological Corridor for large mammals in Panama: comparing multi-species occupancy to movement models

**DOI:** 10.1186/s40462-020-00211-z

**Published:** 2020-05-26

**Authors:** Ninon F. V. Meyer, Ricardo Moreno, Rafael Reyna-Hurtado, Johannes Signer, Niko Balkenhol

**Affiliations:** 1 Departamento de Conservación de la Biodiversidad, El Colegio de la Frontera Sur, Lerma, Campeche, Mexico; 2 Wildlife Sciences, Faculty of Forest Sciences, University of Göttingen, Göttingen, Germany; 3 Fundación Yaguará Panamá, Ciudad del Saber, Panama; 4 Smithsonian Tropical Research Institute, Balboa, Ancón Panama

**Correction to: Mov Ecol (2020) 8:3**

**https://doi.org/10.1186/s40462-019-0186-0**

Following publication of the original article [1], the authors identified an error in the second equation in the ‘Estimating the resistance’ section due to a typesetting mistake: 9 should be replaced by 99. The correct equation is given below and the original article has been corrected.

$$ R=100-99\frac{\left(1-{e}^{\left(-c\ast HS\right)}\right)}{\left(1-{e}^{-c}\right)} $$

The publisher apologises to the authors and readers for the inconvenience.
