# Supplementary material for: Towards the restoration of the Mesoamerican Biological Corridor for large mammals in Panama: comparing multi-species occupancy to movement models
Source: Mov Ecol. 2020 Jan 9;8:3. doi: 10.1186/s40462-019-0186-0 (PMC6953263; doi:10.1186/s40462-019-0186-0)
Supplement: Supplementary file 1 — Additional file 1. Methods - Locations of camera traps used to conduct occupancy modelling across Panama. [file 40462_2019_186_MOESM1_ESM.docx]

**Additional file 1.** Locations of camera traps used to conduct occupancy modeling across Panama. From Meyer et al. (1).

**
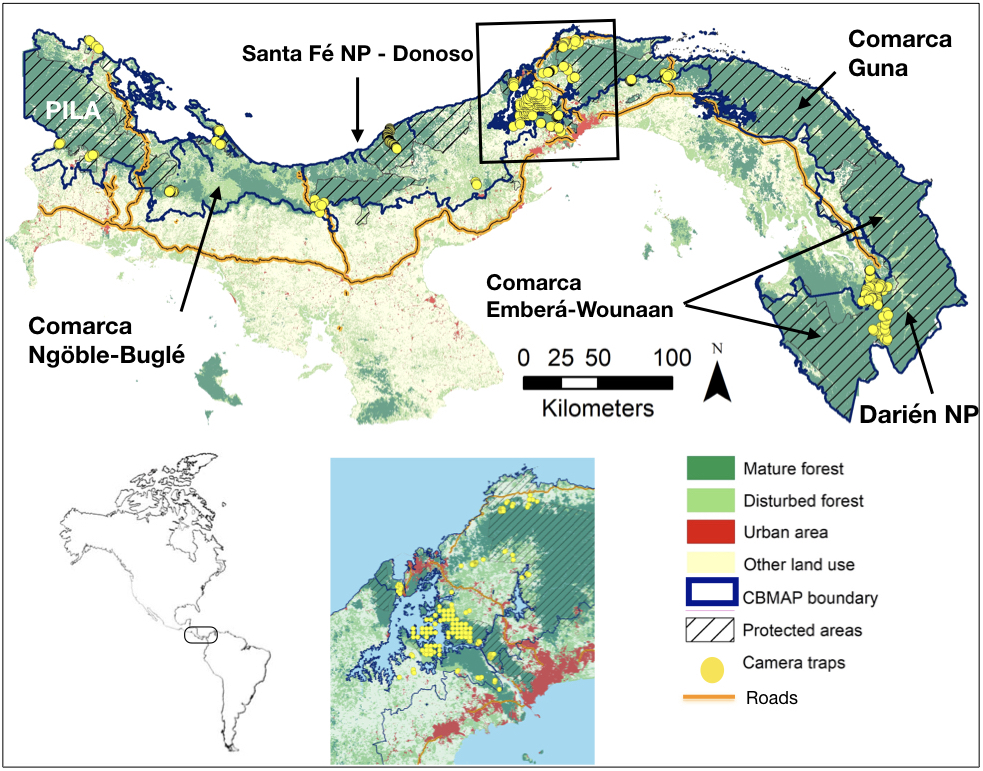
**
