# Supplementary material for: Towards the restoration of the Mesoamerican Biological Corridor for large mammals in Panama: comparing multi-species occupancy to movement models
Source: Mov Ecol. 2020 Jan 9;8:3. doi: 10.1186/s40462-019-0186-0 (PMC6953263; doi:10.1186/s40462-019-0186-0)
Supplement: Supplementary file 2 — Additional file 2. Methods - Environmental variables and selection of characteristic scale. [file 40462_2019_186_MOESM2_ESM.docx]

**Additional file 2.** Environmental variables and selection of characteristic scale

We modeled the influence of six environmental covariates (i.e., anthropogenic and landscape variables) on the probability of occupancy, or suitability for movement. Variables were chosen on the basis of our expertise and information from the literature, and included: (I) distance to nearest road (road), (II) density of human settlements (village), (III) elevation, (IV) percent tree cover (FCOV) in 2012, (V) forest loss (loss) in 2012, and (VI) negative distance within protected areas (DWPA) as a measure of remoteness. For points (camera stations or relocation data) placed outside a protected area, the DWPA was set to 0. Protective status was taken from the National System of Protected Areas (SINAP in Panama). We also assigned protection to the comarca Guna and the comarca Emberá-Wounaan because these territories are under care of indigenous peoples, have a high level of protection, and are mostly covered by old growth forest (2,3). Percent tree cover and forest loss were obtained from (4), and all other layers were obtained from the Ministry of Environment of Panama (MiAmbiente). We generated raster layers of 30 m-resolution in ArcMap (v.10.3.01 ESRI, Redlands, California). We centered and scaled the covariates (5).

Habitat use may not only depend on site-specific characteristics but also on the characteristics of the landscape surrounding a site (6). In other words, animals may respond to different environmental features at different scales, and using a single scale may result in inaccurate estimates of occupancy (7,8). Since we had no a priori knowledge about the scale at which species respond to environmental heterogeneity, we used a univariate analysis to identify the characteristic scale, i.e., the strongest scale of response, for two of our variables separately. We used village as a proxy for hunting pressure, and modelled its impact on the occupancy of mammals within a radius of 5, 10 and 20 km around each sampling point as mammal populations tend to be depleted up to at least 20 kilometers from hunters’ access points (e.g., human settlements, 9). For FCOV, we modelled the proportion of forest within a radius of 150m, 500m, 1 km and 2 km around each camera point. We also tested several thresholds of minimum percentage tree cover that is to be considered ‘forest’ with the Global Forest Change (GFC) layers from Hansen et al. (4). To assign the threshold at a correct spatial scale of response for the modelling, we tested three different proportion values of forest cover, i.e., 50%, 75% and 90 %.

Finally, to avoid multicollinearity (defined as rho > |0.6|), we performed a Spearman correlation test.
