# Supplementary material for: Towards the restoration of the Mesoamerican Biological Corridor for large mammals in Panama: comparing multi-species occupancy to movement models
Source: Mov Ecol. 2020 Jan 9;8:3. doi: 10.1186/s40462-019-0186-0 (PMC6953263; doi:10.1186/s40462-019-0186-0)
Supplement: Supplementary file 3 — Additional file 3. Methods - Transformation curves used to translate habitat suitability values into landscape resistance values. [file 40462_2019_186_MOESM3_ESM.docx]

**Additional file 3.** Transformation curves to translate habitat suitability values into resistance values. Following Trainor et al. (10), we used a linear transformation (continuous line):

R = 100 – (100 * HS)

and two negative exponential functions (dashed lines):

R = 100 – 99 * $\frac{(1-e^{\left( -c*HS \right)})}{(1-e^{\left( -c \right)})}$

where R is the resistance, HS is the habitat suitability (i.e., the occupancy probability ψ, or the prediction of movement S as derived from SSF), and the factor c (3; 8) determines the shape of the curve.
