# Supplementary material for: Towards the restoration of the Mesoamerican Biological Corridor for large mammals in Panama: comparing multi-species occupancy to movement models
Source: Mov Ecol. 2020 Jan 9;8:3. doi: 10.1186/s40462-019-0186-0 (PMC6953263; doi:10.1186/s40462-019-0186-0)
Supplement: Supplementary file 4 — Additional file 4. Methods- Probability of occupancy and proportion of the study area. [file 40462_2019_186_MOESM4_ESM.docx]

**Additional file 4.** We plotted the probability of occupancy against the proportion of the study area (A ). We identified the occupancy threshold where the slope was the highest (A), and used this occupancy value as the threshold to identify areas where occupancy was at least equivalent or higher to that value (B). The proportion of area which was considered suitable and which we hence sought to connect was larger for the tolerant group (50% of the study area; 8 core areas; ψ = 0.2 ) than for the sensitive group (area = 40%; 6 core areas; ψ= 0.3). Psi = ψ and is shown as %.

**
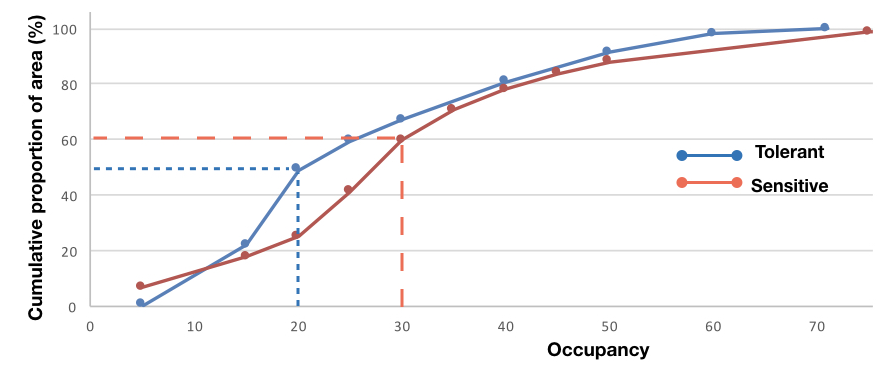
**

A

**
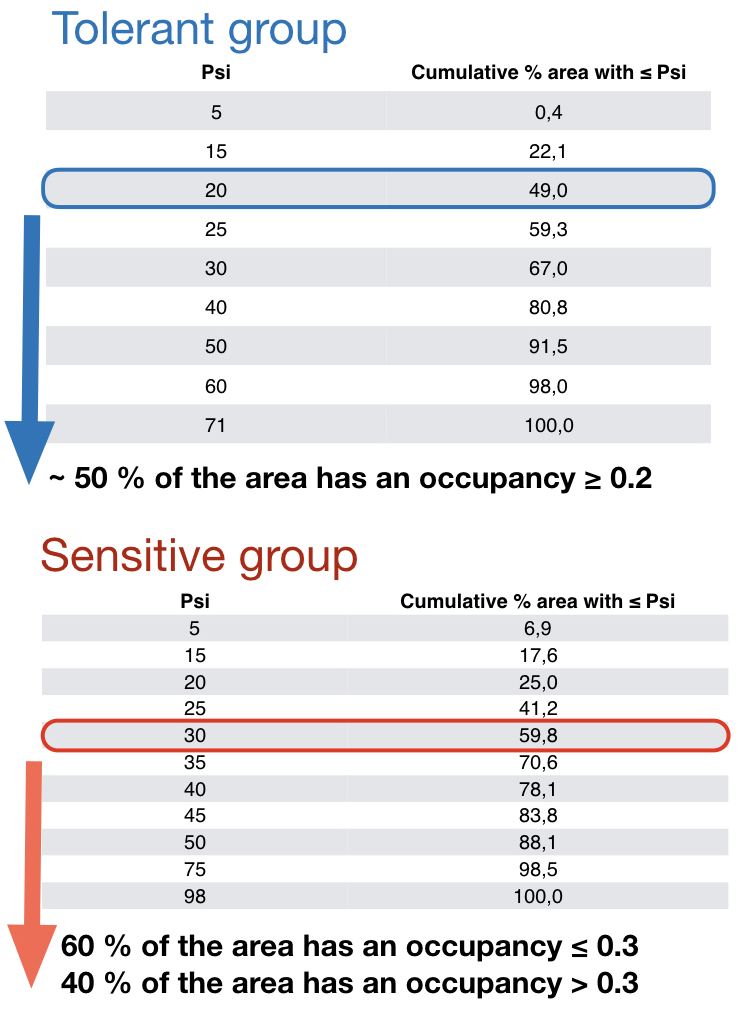
**

B
