# Supplementary material for: Towards the restoration of the Mesoamerican Biological Corridor for large mammals in Panama: comparing multi-species occupancy to movement models
Source: Mov Ecol. 2020 Jan 9;8:3. doi: 10.1186/s40462-019-0186-0 (PMC6953263; doi:10.1186/s40462-019-0186-0)

**Additional file 5.** Panama with core areas among which we sought to estimate functional connectivity and identify effective corridors for two groups of species which were considered tolerant (pink in upper map) and sensitive (yellow in lower map). Core areas were identified based on an occupancy probability (Psi) threshold of ≥ 0.2 for the tolerant species, and Psi ≥ 0.3 for the sensitive species. Protected areas within our study area are outlined in black and generally overlapped with the core areas identified. However, some core areas of tolerant species go beyond the limits of protected areas, or are not protected at all (pink area that is not delimited by a black line). Conversely, not all protected areas were considered core areas for sensitive species, as many core areas, especially in the western part of Panama were shrinked compared to the limits of the protected areas.


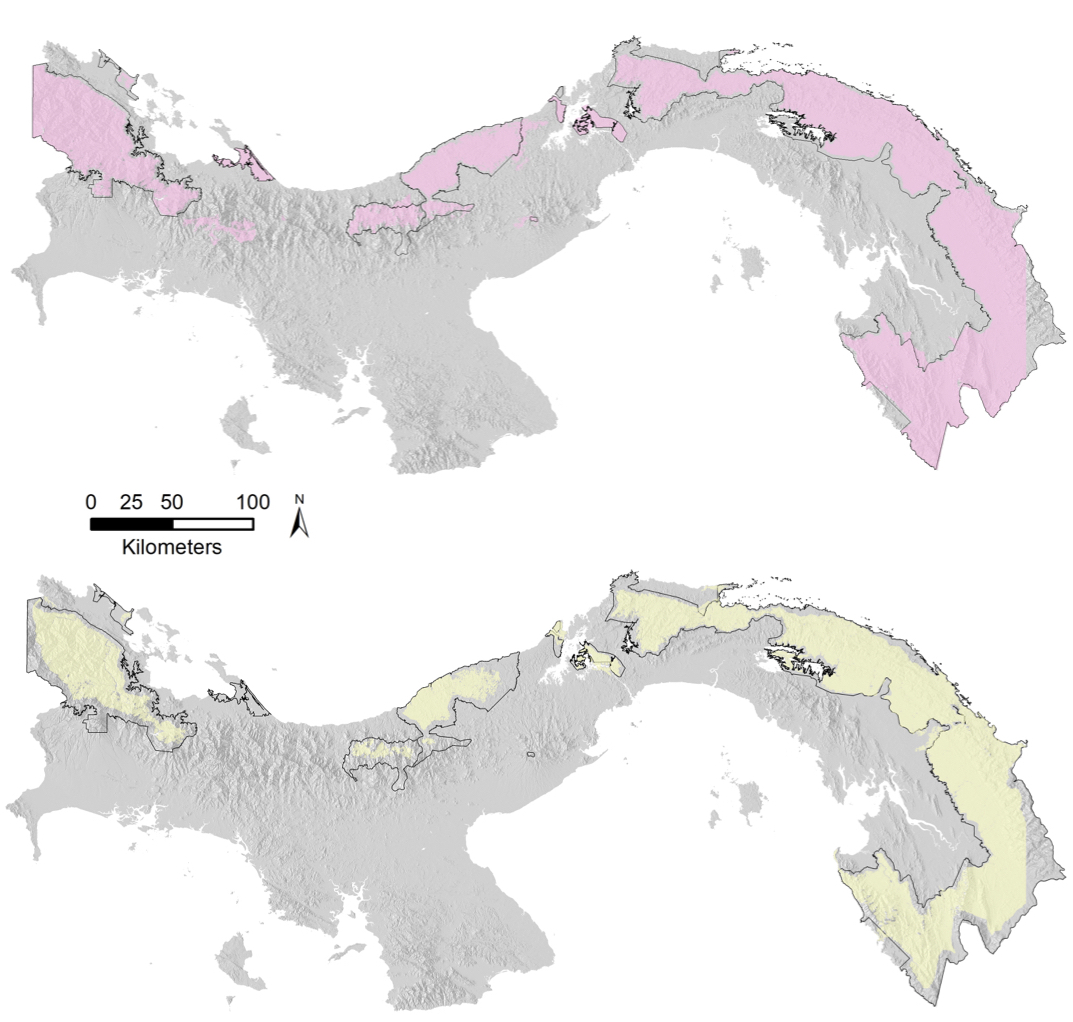

Supplement: Supplementary file 5 — Additional file 5. Methods - Core areas of two groups of species. [file 40462_2019_186_MOESM5_ESM.docx]
