# Supplementary material for: Towards the restoration of the Mesoamerican Biological Corridor for large mammals in Panama: comparing multi-species occupancy to movement models
Source: Mov Ecol. 2020 Jan 9;8:3. doi: 10.1186/s40462-019-0186-0 (PMC6953263; doi:10.1186/s40462-019-0186-0)
Supplement: Supplementary file 6 — Additional file 6. Results - GPS relocations and steps for each individual. [file 40462_2019_186_MOESM6_ESM.docx]

**Additional file 6.** Number of GPS relocations and steps derived for each individual

|  | GPS locations | Steps-All | Steps-Travel |
| --- | --- | --- | --- |
| **TOLERANT** |  |  |  |
| *Ocelot* | 1068 | 695 | 349 |
| *Puma* | 1614 | 1616 | 410 |
| **SENSITIVE** |  |  |  |
| *White-lipped peccary 1* | 2127 | 1978 | 821 |
| *White-lipped peccary 2* | 280 | 253 | 88 |
| *White-lipped peccary 3* | 993 | 867 | 224 |
