# Supplementary material for: Towards the restoration of the Mesoamerican Biological Corridor for large mammals in Panama: comparing multi-species occupancy to movement models
Source: Mov Ecol. 2020 Jan 9;8:3. doi: 10.1186/s40462-019-0186-0 (PMC6953263; doi:10.1186/s40462-019-0186-0)
Supplement: Supplementary file 7 — Additional file 7. Results - Determination of the optimal scale for forest cover, forest loss, and density of villages. [file 40462_2019_186_MOESM7_ESM.docx]

**Additional file 7**. Optimal scales determined for forest cover (FCOV), forest loss (loss) and density of villages (village), and which were identified via a univariate analysis for each combination of individual and movement mode.

|  | **Mode** | **FCOV** | **loss** | **village** |
| --- | --- | --- | --- | --- |
| **OCCUPANCY** |  | fcov75_150m | na | village_10km |
| **SSF - TOLERANT** |  |  |  |  |
| *Ocelot* | SSF-All | fcov_150m | loss_150m | Village_10km |
|  | SSF-Travel | fcov_150m | loss_1km | village_2km |
| *Puma* | SSF-All | fcov_150m | loss_2km | village_20km |
|  | SSF-Travel | fcov_150m | loss_2km | village_20km |
| **SSF - SENSITIVE** |  |  |  |  |
| *WLP1* | SSF-All | fcov75_500m | loss_1km | village_2km |
|  | SSF-Travel | fcov_1km | loss_500m | village_2km |
| *WLP2* | SSF-All | Fcov90_1km | loss_500m | village_5km |
|  | SSF-Travel | fcov_1km | loss_500m | village_10km |
| *WLP3* | SSF-All | fcov_150 | loss_150m | village_5km |
|  | SSF-Travel | fcov_150 | loss_500m | village_5km |
