# Supplementary material for: Towards the restoration of the Mesoamerican Biological Corridor for large mammals in Panama: comparing multi-species occupancy to movement models
Source: Mov Ecol. 2020 Jan 9;8:3. doi: 10.1186/s40462-019-0186-0 (PMC6953263; doi:10.1186/s40462-019-0186-0)
Supplement: Supplementary file 8 — Additional file 8. Results - Species-specific coefficients for environmental variables estimated with occupancy modeling. [file 40462_2019_186_MOESM8_ESM.docx]

**Additional file 8**. Species-specific coefficients for variables based on an occupancy model with detection probability that was allowed to vary with sampling effort. See Meyer et al. (1) for 95% CRI, median and sd.

| **Covariates** | **JAG** | **PUM** | **OCE** | **WTD** | **RBD** | **TAP** | **WLP** | **SAI** | **ANT** |
| --- | --- | --- | --- | --- | --- | --- | --- | --- | --- |
| **Occupancy** | |  |  |  |  |  |  |  |  |
| α (constant) | -1.64 | -0,5 | 0.02 | -2.04 | -0.75 | -1.34 | -2.59 | 0.09 | -1.37 |
| road | 0.01 | -0.02 | 0.30 | 0.41 | -0.04 | -0.08 | 0.07 | 0.27 | -0.10 |
| FCOV | -0.22 | -0.17 | 0.008 | 0.58 | 0.23 | 0.33 | 0.39 | 0.13 | 0.11 |
| DWPA | -0.22 | 0.16 | -0.22 | -0.56 | -0.28 | -0.26 | -0.49 | -0.37 | -0.12 |
| village | -0.07 | 0.08 | 0.20 | -0.21 | 0.10 | 0.12 | 0.003 | -0.08 | -0.19 |
| elevation | -0.10 | -0.02 | 0.06 | 0.12 | 0.15 | 0.04 | -0.14 | -0.03 | -0.12 |

Species abbreviations: JAG = jaguar; PUM = puma; OCE = ocelot ; WTD = white-tailed deer; RBD= red brocket deer; TAP = tapir; WLP= white-lipped peccary; SAI = collared peccary; ANT = giant anteater.
