# Supplementary material for: Towards the restoration of the Mesoamerican Biological Corridor for large mammals in Panama: comparing multi-species occupancy to movement models
Source: Mov Ecol. 2020 Jan 9;8:3. doi: 10.1186/s40462-019-0186-0 (PMC6953263; doi:10.1186/s40462-019-0186-0)
Supplement: Supplementary file 9 — Additional file 9. Results - AICc of SSF models for each individual. [file 40462_2019_186_MOESM9_ESM.docx]

**Additional file 9**. Corrected Aikaike Information Criterion scores (AICc) of step selection functions modeled with uncorrelated variables for each individual, and using all relocation data (SSF-All) or traveling relocation data (SSF-Travel). Models with the lowest AICc had the highest support.

|  | Mode | Model | AICc |
| --- | --- | --- | --- |
| **TOLERANT** |  |  |  |
| *Ocelot* | SSF-All | **FCOV + loss + village + DWPA + elevation + road** | **6336** |
|  | SSF-Travel | **loss + DWPA + village + elevation + FCOV**  road + DWPA + village + elevation + FCOV | **3111**  3113 |
| *Puma* | SSF-All | **elevation + loss + FCOV + DWPA**  road + loss + FCOV + DWPA  village + loss + FCOV + DWPA | **8967**  8970  8970 |
|  | SSF-Travel | **DWPA + FCOV + loss + elevation**  DWPA + FCOV + loss + road  FCOV + loss + village | **3227**  3234  3240 |
| **SENSITIVE** |  |  |  |
| *WLP1* | SSF-All | **village + loss + FCOV + elevation + road**  DWPA + village + loss + FCOV | **18212**  18251 |
|  | SSF-Travel | **elevation + road + loss + FCOV + village**  DWPA + loss + FCOV + village | **7546**  7566 |
| *WLP2* | SSF-All | **DWPA + loss + FCOV**  road + loss + FCOV  DWPA + village + FCOV  FCOV + elevation + loss | **2333**  2336  2336  2337 |
|  | SSF-Travel | **village + loss + FCOV**  road + loss + FCOV  DWPA + loss + FCOV  elevation + loss + FCOV | **804**  807  808  810 |
| *WLP3* | SSF-All | **village + loss + FCOV + DWPA + elevation**  village + loss + FCOV + road + elevation | **7961**  7963 |
|  | SSF-Travel | **elevation + DWPA + FCOV + loss**  FCOV + loss + road + village + elevation | **2052**  2054 |

WLP = white-lipped peccary; FCOV = forest cover within a certain radius of the relocations; loss = forest loss; village = density of villages within a certain radius of the relocations; DWPA = distance within protected area.

**References**

1. Meyer NFV., Moreno R, Sutherland C, la Torre JA, Esser HJ, Jordan CA, et al. Effectiveness of Panama as an intercontinental land bridge for large mammals. Conserv Biol. 2019;0(0):1–13.

2. Vergara-Asenjo G, Potvin C. Forest protection and tenure status: The key role of indigenous peoples and protected areas in Panama. Glob Environ Chang. 2014;28(1):205–15. Available from: http://dx.doi.org/10.1016/j.gloenvcha.2014.07.002

3. IUCN-ORMACC. Map of the Indigenous peoples, protected areas and natural ecosystem of Central America. 2016; Available from: ormacc@iucn.org

4. Hansen MCC, Potapov P V, Moore R, Hancher M, Turubanova SA a, Tyukavina A, et al. High-Resolution Global Maps of 21st-Century Forest Cover Change. Science (80). 2013;342(November):850–4. Available from: http://www.ncbi.nlm.nih.gov/pubmed/24233722

5. Schielzeth H. Simple means to improve the interpretability of regression coefficients. Methods Ecol Evol. 2010;103–13.

6. De Knegt HJ, Van Langevelde F, Skidmore AK, Delsink A, Slotow R, Henley S, et al. The spatial scaling of habitat selection by African elephants. J Anim Ecol. 2011;80(1):270–81.

7. Zeller KA, McGarigal K, Whiteley AR. Estimating landscape resistance to movement: A review. Landsc Ecol. 2012;27(6):777–97.

8. Zeller KA, McGarigal K, Beier P, Cushman SA, Vickers TW, Boyce WM. Sensitivity of landscape resistance estimates based on point selection functions to scale and behavioral state: Pumas as a case study. Landsc Ecol. 2014;29(3):541–57.

9. Benítez-López A, Alkemade R, Schipper AM, Ingram DJ, Verweij PA, Eikelboom JAJ, et al. The impact of hunting on tropical mammal and bird populations. Science (80). 2017;356(6334):180–3.

10. Trainor AM, Walters JR, Morris WF, Sexton J, Moody A. Empirical estimation of dispersal resistance surfaces: A case study with red-cockaded woodpeckers. Landsc Ecol. 2013;28(4):755–67.
