# Supplementary material for: Towards the restoration of the Mesoamerican Biological Corridor for large mammals in Panama: comparing multi-species occupancy to movement models
Source: Mov Ecol. 2020 Jan 9;8:3. doi: 10.1186/s40462-019-0186-0 (PMC6953263; doi:10.1186/s40462-019-0186-0)

Additional file 11. Output maps of the connectivity analysis showing multi-species connectivity scenarios between core areas of Panama. Corridors were modeled on the basis of varying (1) data type (camera trap and GPS telemetry) and modeling approach (occupancy and step selection functions), (2) species that were divided into two groups (tolerant: jaguar, puma, ocelot, white-tailed deer, red brocket deer, collared peccary, and sensitive: Baird's tapir, white-lipped peccary, giant anteater), and (3) transformation curve to translate habitat suitability into resistance (one negative linear, and two negative exponential).

Effect of data type and modeling approach

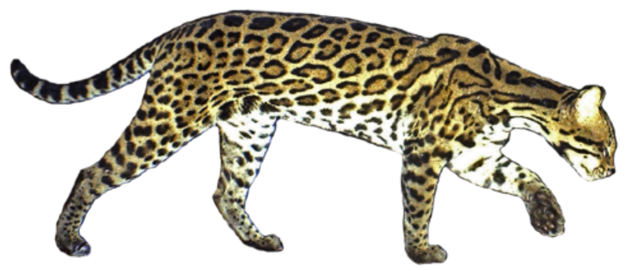

Tolerant

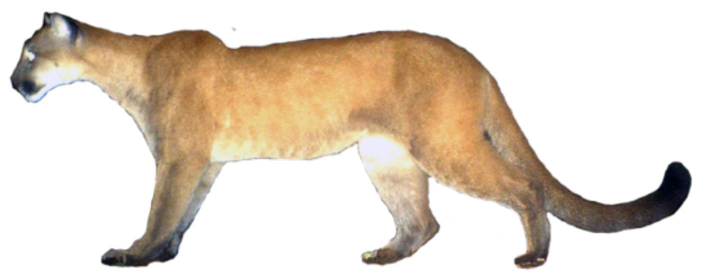

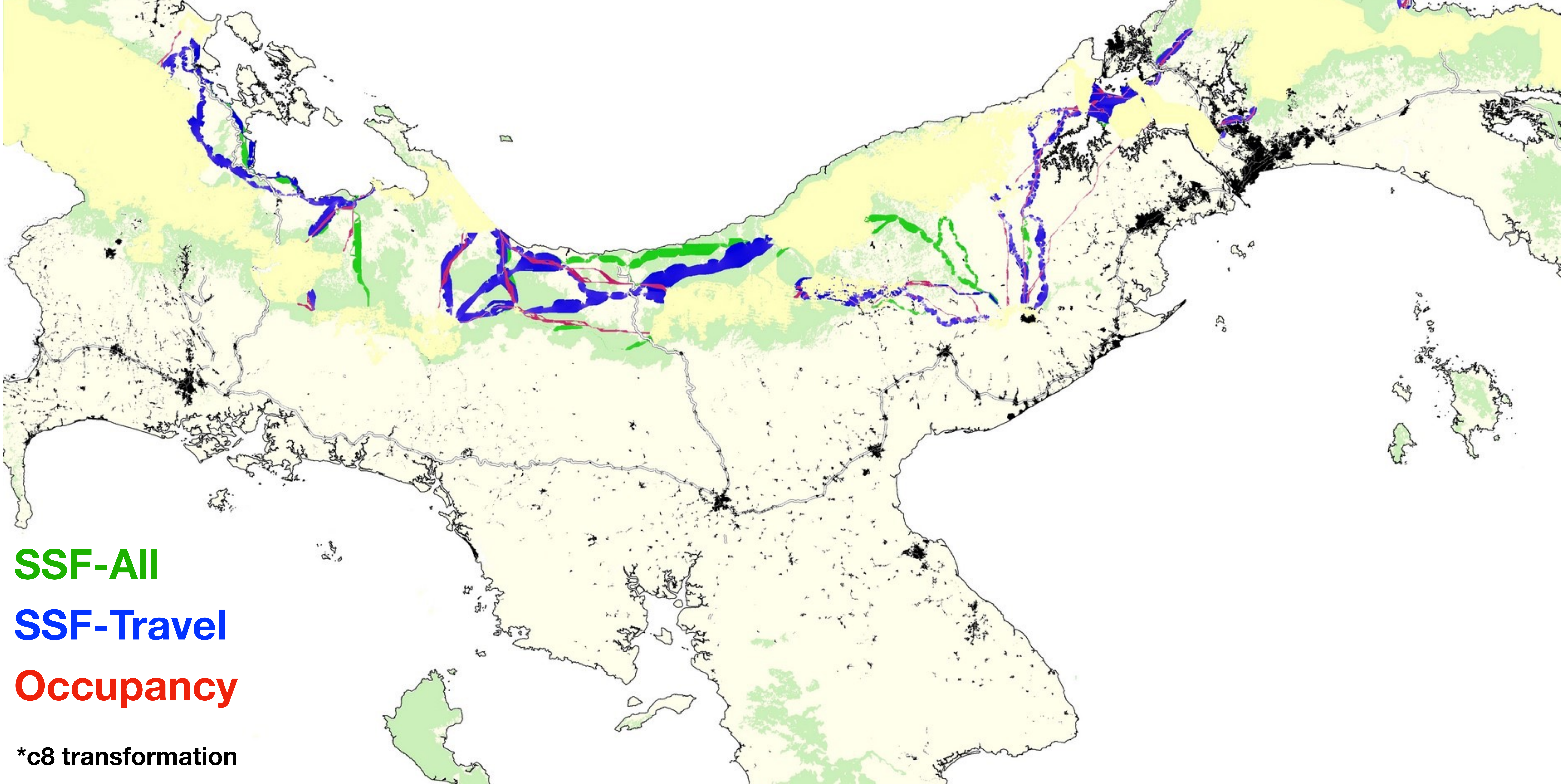

Sensitive

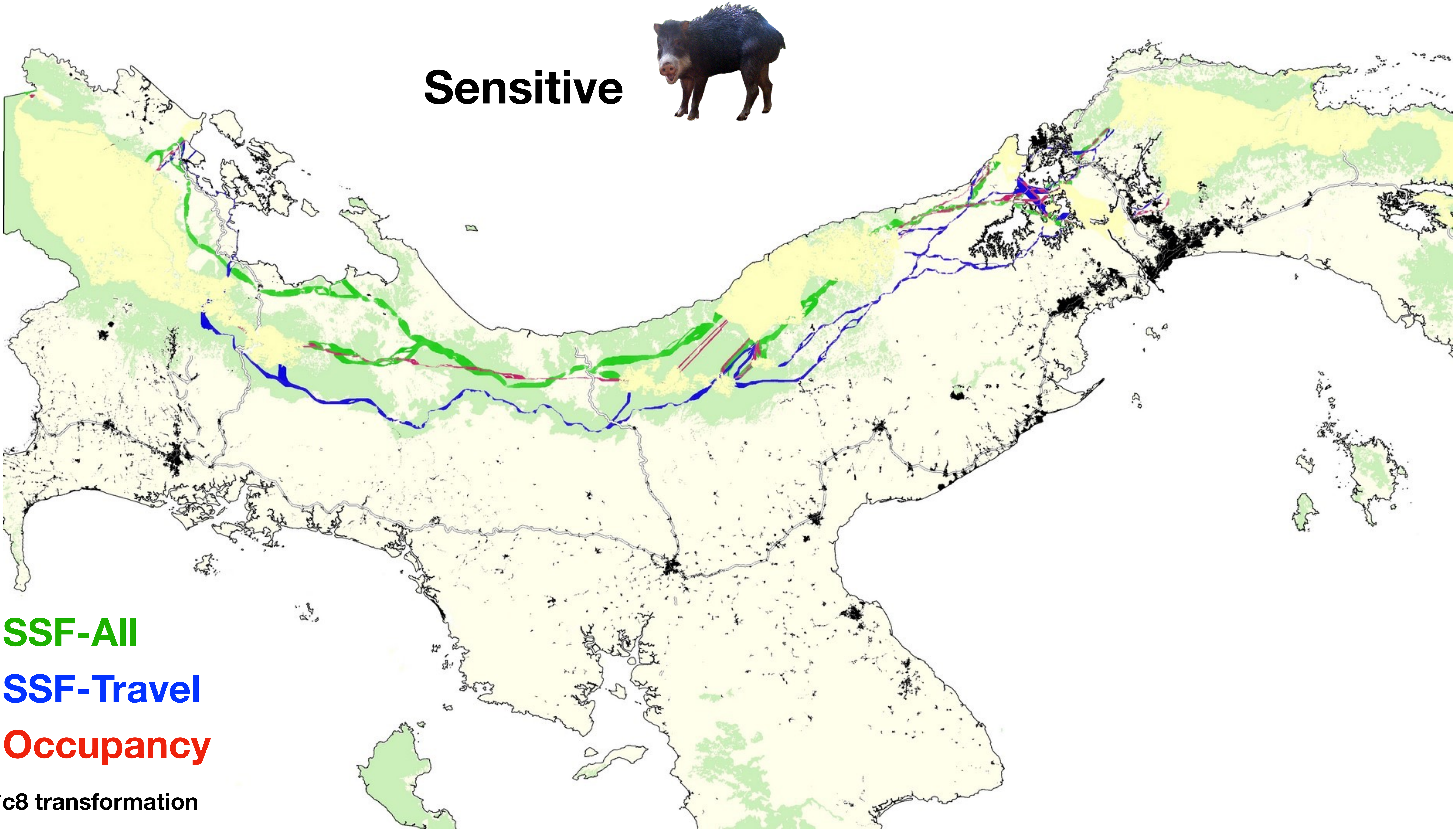

SSF-All

SSF-Travel

Occupancy

*c8 transformation

Effect of group of species

SSF-All

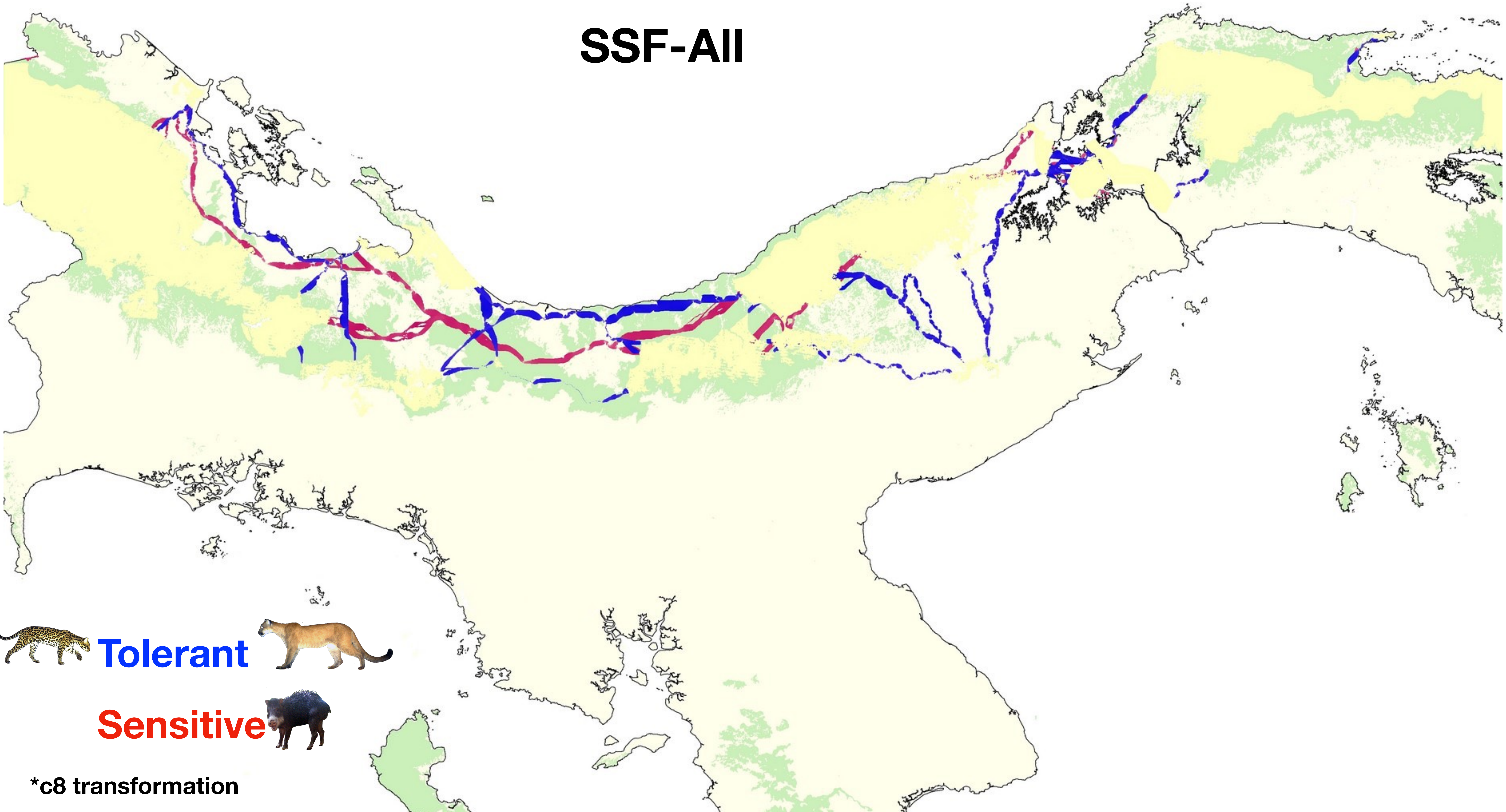

*c8 transformation

SSF-Travel

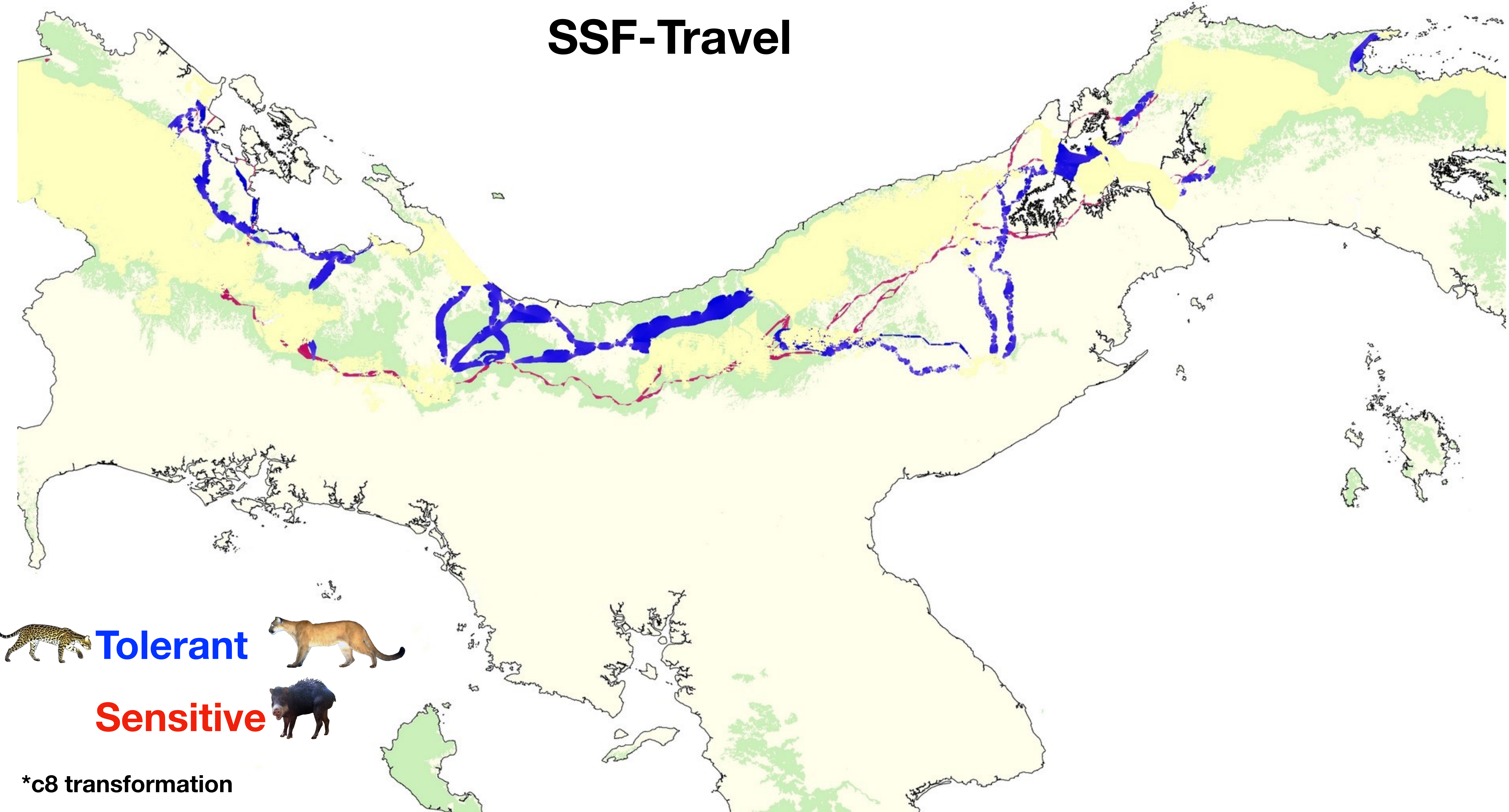

Occupancy

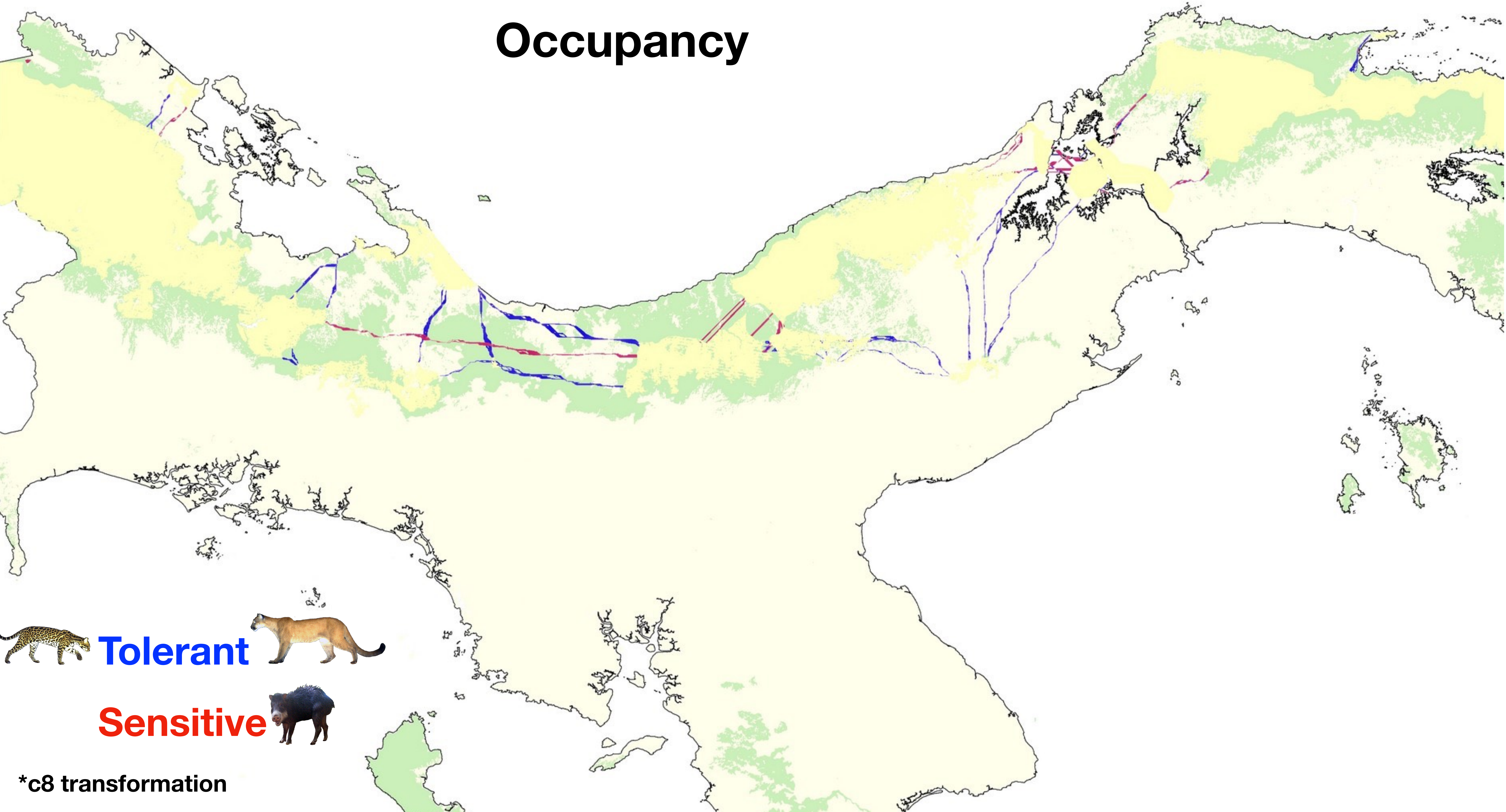

Effect of transformation

from suitability to resistance

Tolerant - SSF-All

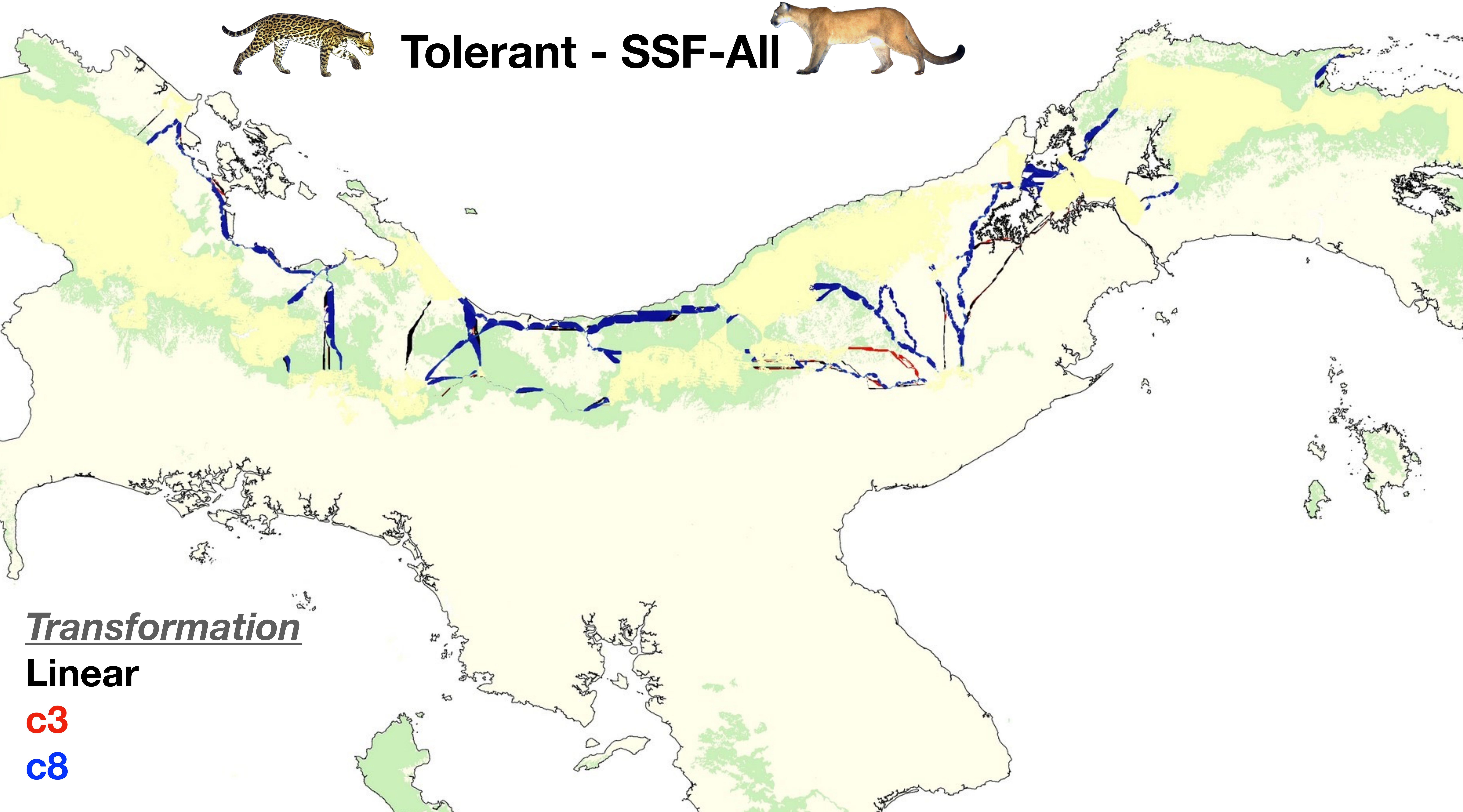

Transformation
Linear
c3
c8

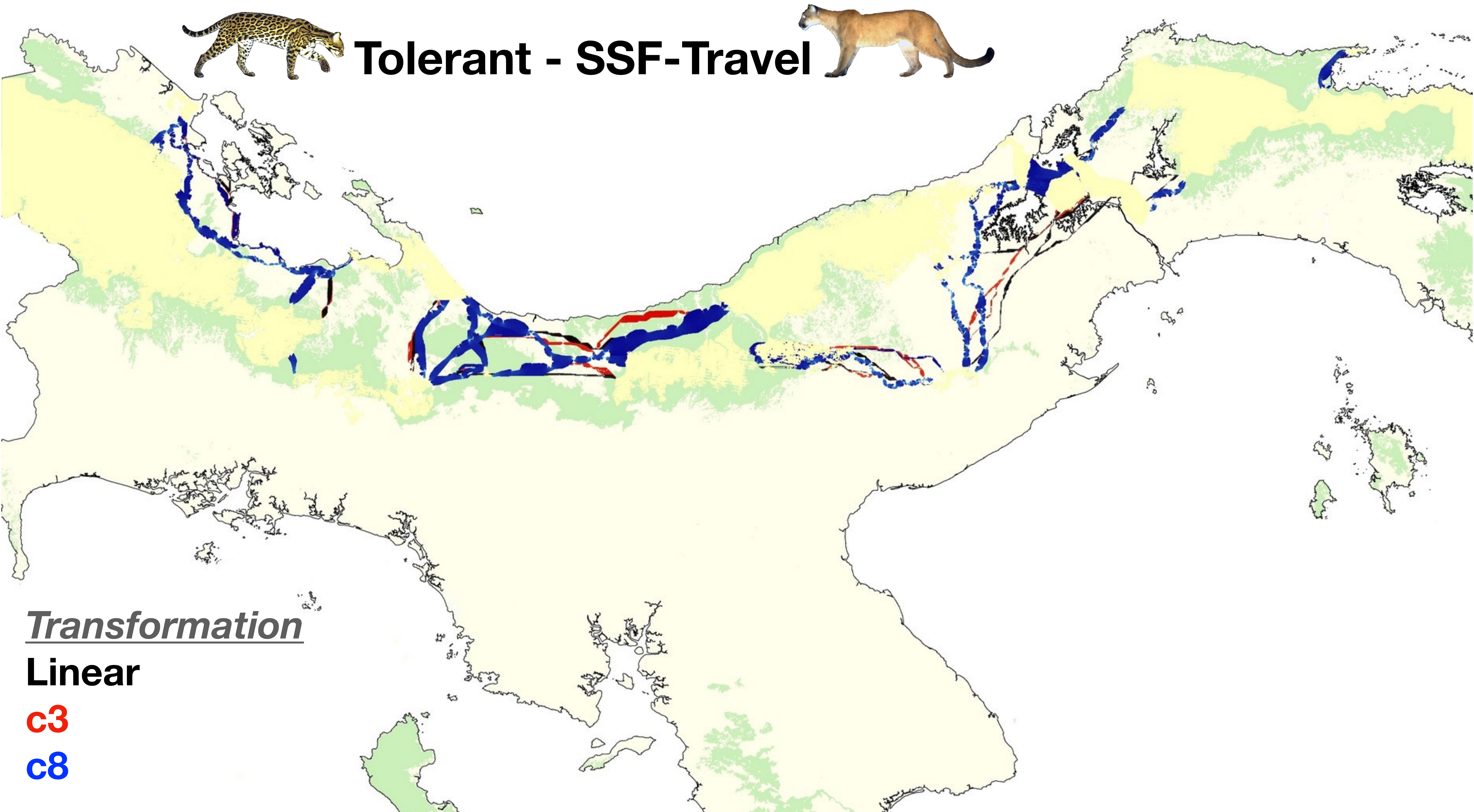
Tolerant - SSF-Travel
Transformation
Linear
c3
c8

Tolerant - Occupancy

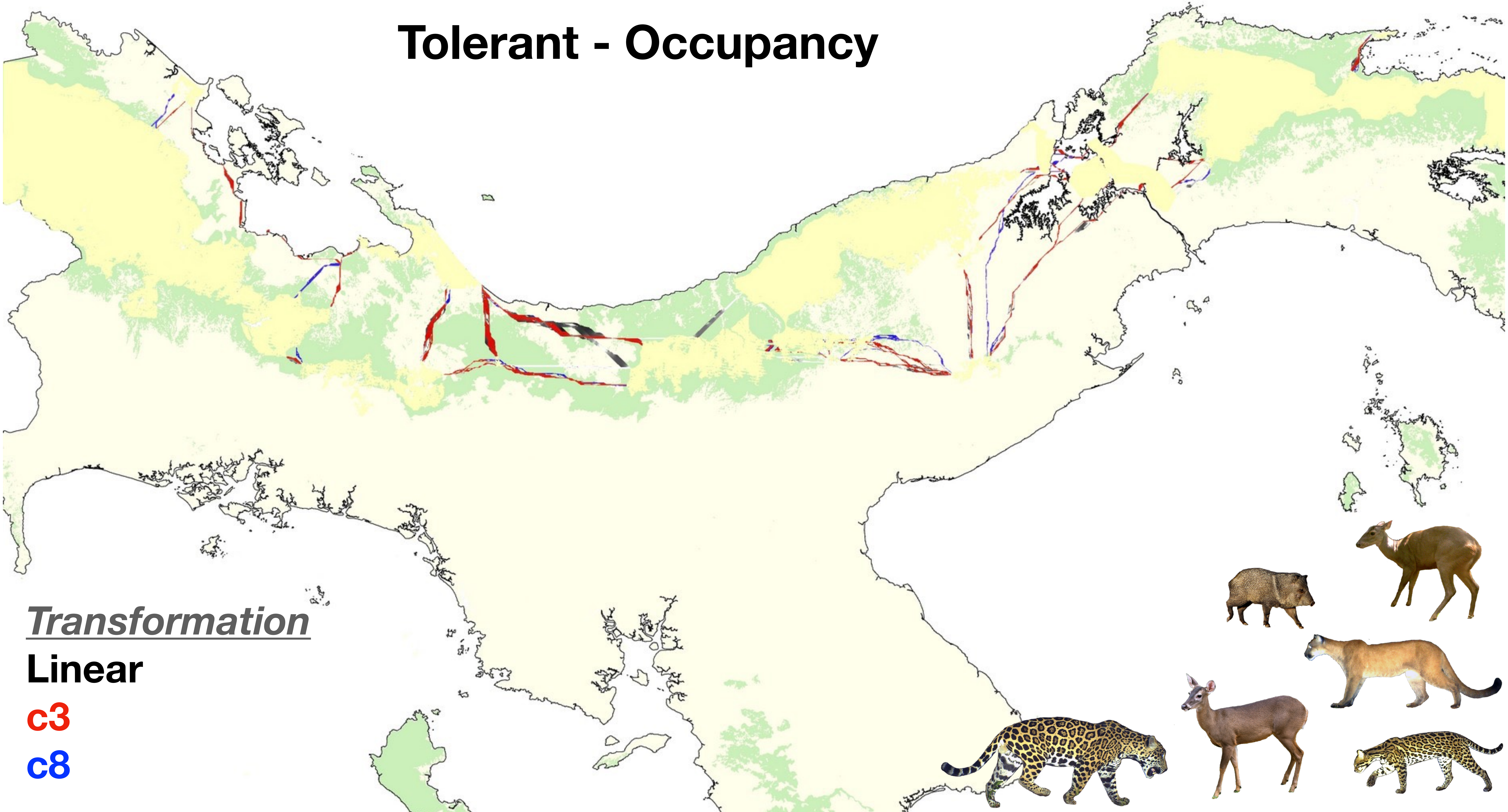

Sensitive - SSF-All

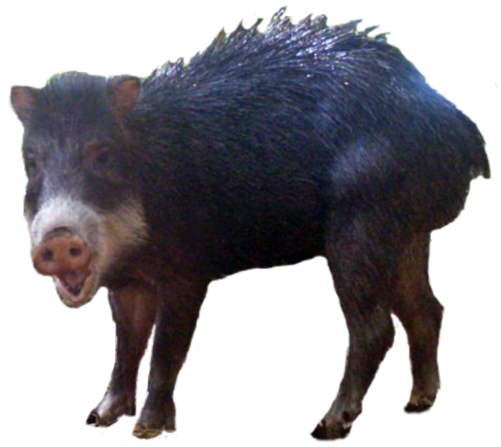

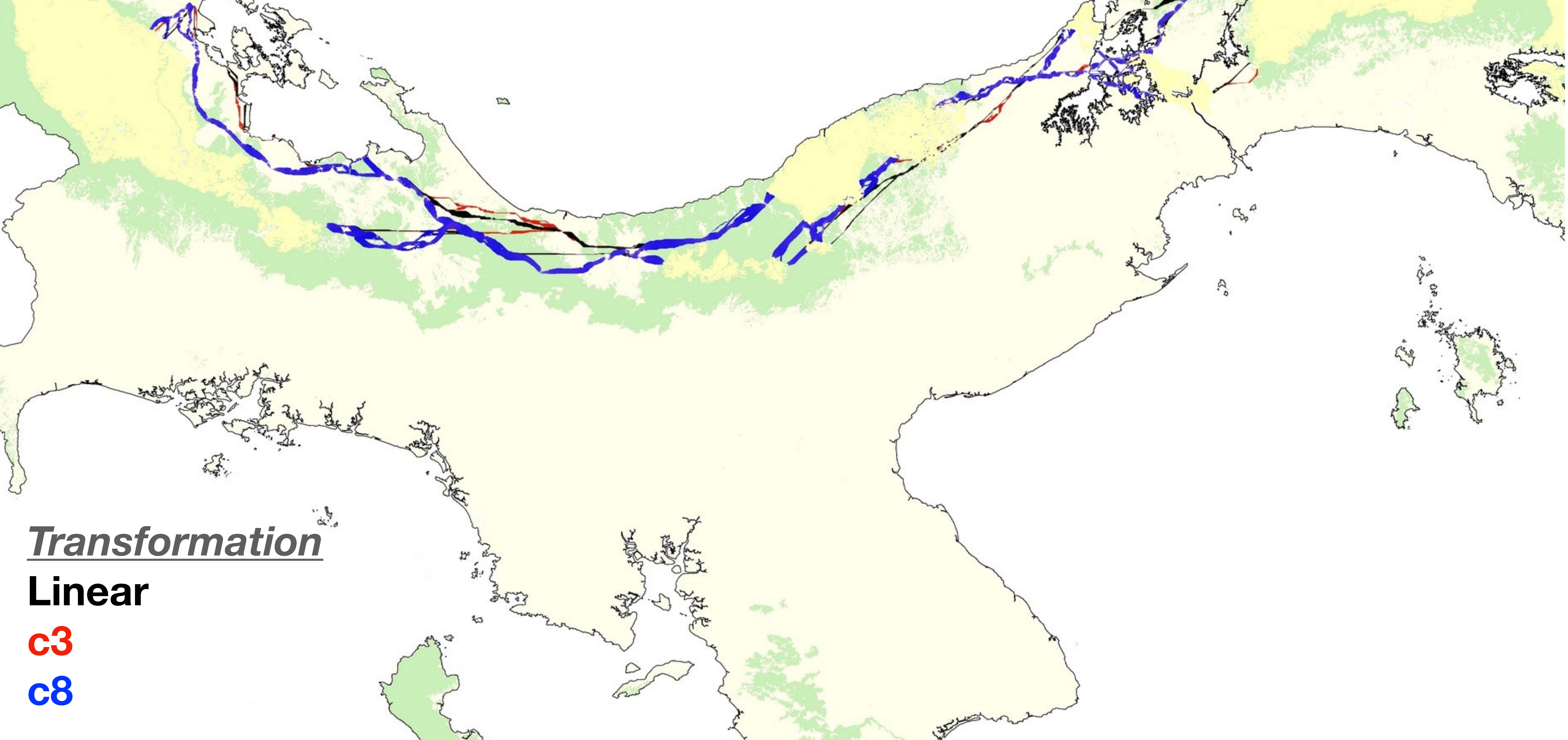

Sensitive - SSF-Travel

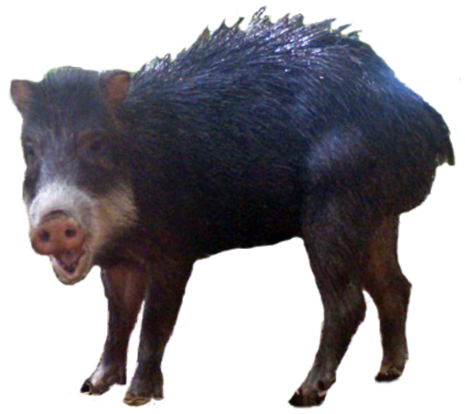

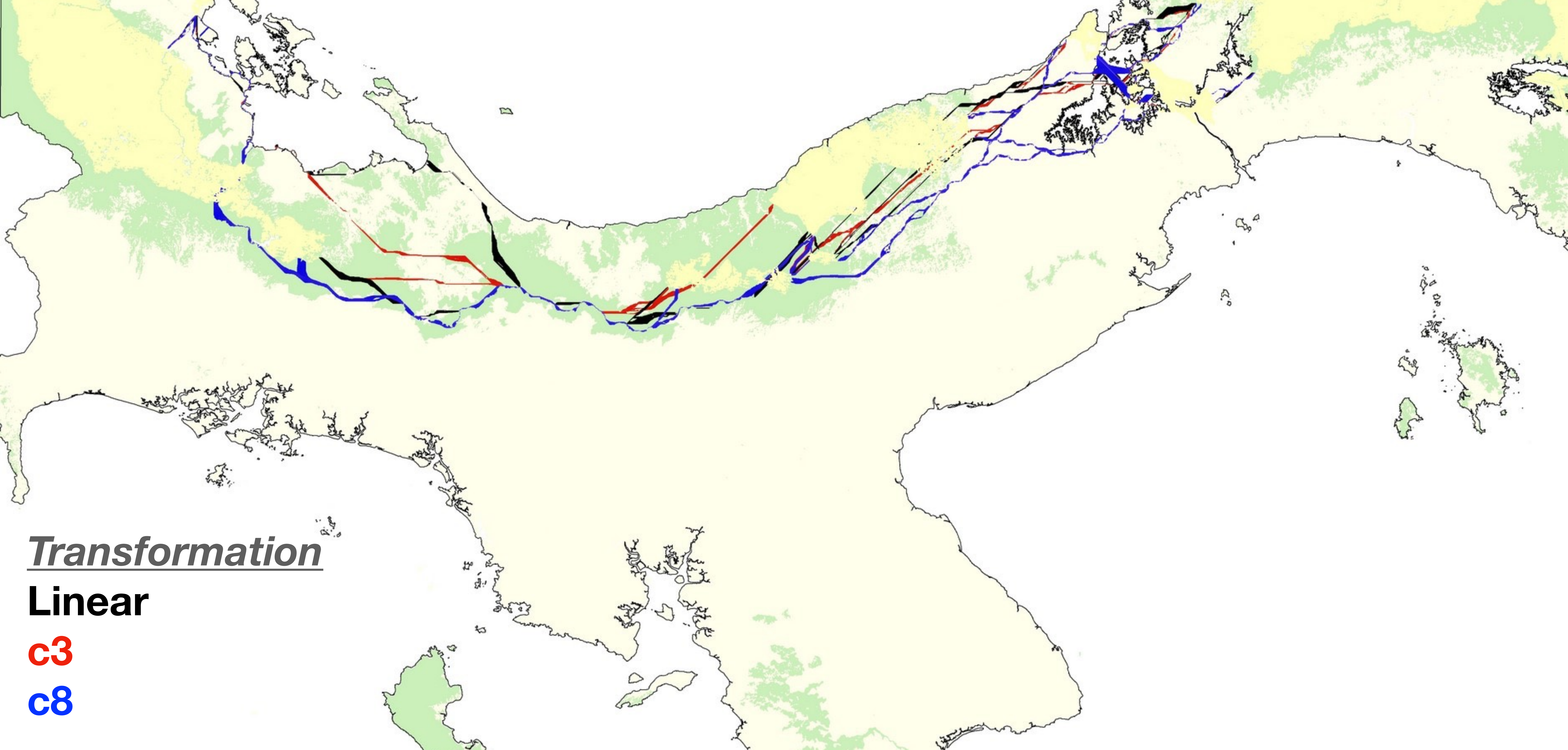

Sensitive - Occupancy

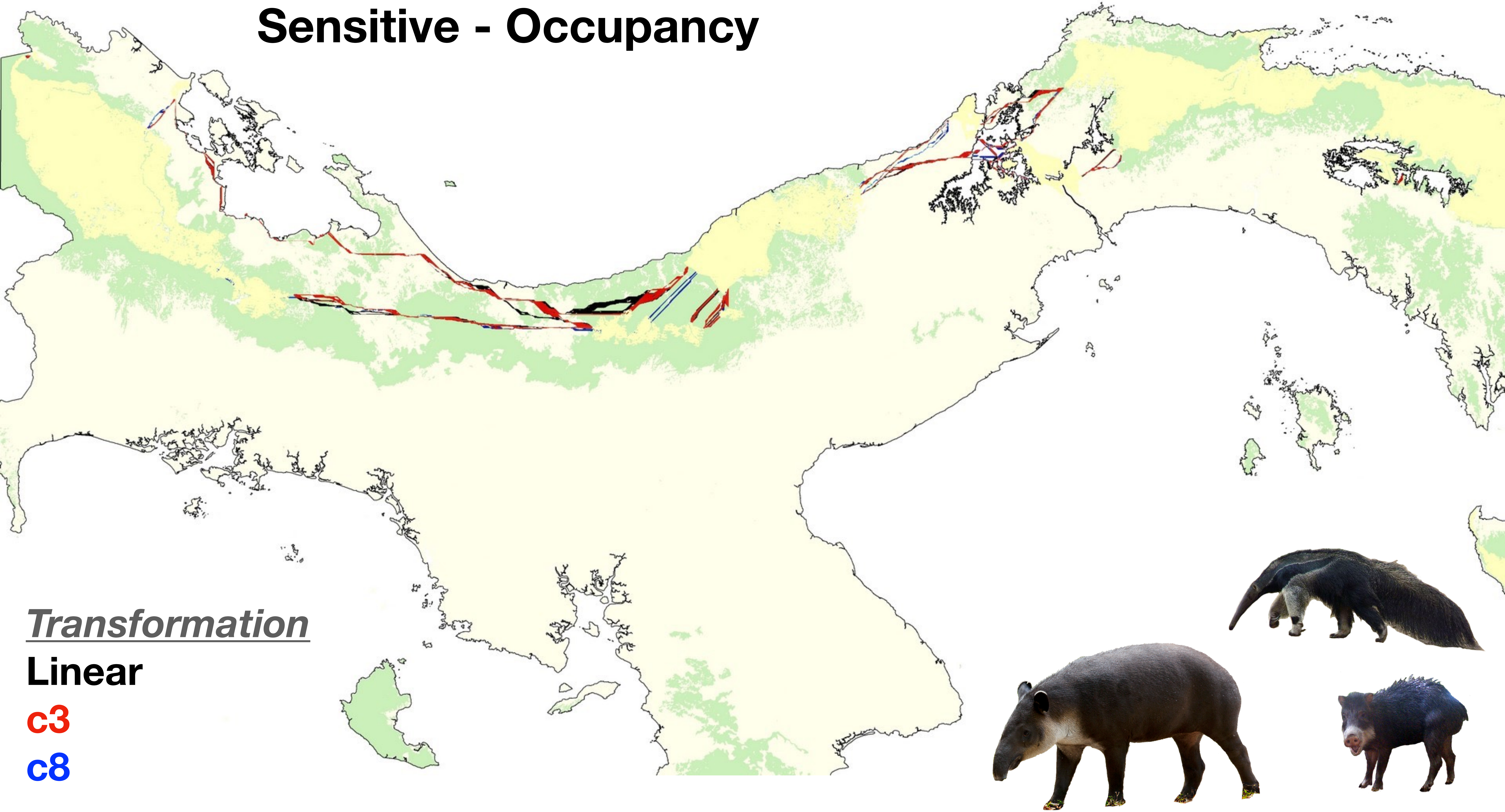

Supplement: Supplementary file 11 — Additional file 11. Results - Maps with multi-species connectivity scenarios. [file 40462_2019_186_MOESM11_ESM.pdf]
